# Content development for a new item-bank for measuring multifocal contact lens performance

**DOI:** 10.1186/s41687-024-00689-w

**Published:** 2024-02-08

**Authors:** Elsa Albero-Ros, Amalia Lorente-Velázquez, Mariano González-Pérez

**Affiliations:** 1 Department of Optometry and Vision, Faculty of Optics and Optometry, Universidad Complutense de Madrid, Arcos de Jalón, 118, 28037 Madrid, Spain; 2 Alain Afflelou Óptico, Portugal, Av. António Augusto de Aguiar, 11, 1050-016 Lisbon, Portugal; 3 Clinical and Experimental Research Group (CEER), Faculty of Optics and Optometry, Universidad Complutense de Madrid, Arcos de Jalón, 118, 28037 Madrid, Spain

**Keywords:** Presbyopia, Patient-reported outcome, Qualitative research, Content-validity, Item bank

## Abstract

**Background:**

Presbyopia is an age-related condition that causes a decreased ability to focus on nearby objects. Multifocal contact lenses are commonly used to address this issue. However, there seems to be a notable dissatisfaction among multifocal contact lens wearers. The absence of a reliable instrument to measure the patient’s perspective, despite the widespread use of this method, highlights the need for further research in this area.

**Objective:**

The objective of this study is to develop an item-bank integrating all domains necessary to assess the patient’s perspective on multifocal contact lens performance, offering a comprehensive measure. The item-bank will ensure a high level of content validity, be self-administered, and will initially be available in Spanish. The aim of this tool is to serve as a valuable resource for research and optometric clinics, facilitating the follow-up of patients with presbyopia who wear multifocal contact lenses or those who are newly starting to use them.

**Methodology:**

The MCL-PRO item bank, followed a systematic and step-wise inductive approach to gather information, following the recommendations outlined in the COSMIN guidelines and similar studies. The process involved the following steps: (1) Literature review and relevant existing items identification (2) Social media review, (3) Semi-structured focus groups, (4) performing qualitative analysis, (5) refining and revising the items, and (6) generating the content of the item bank.

**Results:**

A total of 575 items were included in the item-bank hosted under 8 different domains that were found to be important for presbyopic population: visual symptoms (213), activity limitation (111), ocular symptoms (135), convenience (36), emotional well-being (33), general symptoms (16), cognitive issues (21) and economic issues (10).

**Conclusion:**

The item-bank created has followed standardised methodology for its development and encloses all the aspects for MCL performance evaluation from patients perspective.

**Supplementary Information:**

The online version contains supplementary material available at 10.1186/s41687-024-00689-w.

## Introduction

Presbyopia is a condition where the ability to focus on nearby objects diminishes with age. It typically starts between the ages of 38–45 and affects everyone by the time they reach 50–52 years old [1]. The prevalence of presbyopia has been increasing in Europe, with the population of individuals aged 65 and over growing from 16 to 21% between 2002 and 2022, according to Eurostat. Various correction methods are used for presbyopia, including progressive, bifocal, or supplementary reading spectacles, intraocular multifocal lenses, and contact lenses (CL) [2, 3]. When it comes to contact lenses, there are three main categories for correcting presbyopia [1]:

1. Supplementary spectacle correction over contact lens.
2. monovision Contact lenses.
3. Multifocal Contact lenses (MCL), specifically designed for individuals with presbyopia.

However, many contact lens wearers discontinue their use when presbyopia develops. Among those who continue, the most common approach is to use supplementary spectacle correction over contact lenses [1]. Only 29% of contact lens users opt for MCL [4], and while reported success rates after three months range from 67 to 83%, dropouts are frequent, resulting in an actual long-term success rate of 30–40% [5]. This can be attributed to inadequate fitting skills, a lack of suitable MCL options [6], and/or a lack of indicators for proper evaluation and patient satisfaction.

Clinical tests have been used to assess the performance of MCL. However, relying solely on initial tests conducted in the clinic is not sufficient for predicting the success of MCL. Therefore, incorporating additional indicators to aid in the selection and evaluation of the most suitable presbyopic lens would be beneficial in reducing dropout rates and minimizing chair time [7]. Regulatory agencies, such as the U.S. Food and Drug Administration and the European Medicines Agency, now require the inclusion of patient-reported outcomes (PROs) in assessing the effectiveness of medical treatments and devices. PROs refer to reports about a patient’s health directly from the patient themselves, without interpretation by clinicians or others [8].

A search conducted in October 2021 yielded 12 trials investigating MCL performance [2, 7, 9–18]. These trials focused on assessing non-clinical outcomes related to MCL, including symptoms, functional limitations, and health perception. Notably, quality of life was not evaluated in these trials, although it is commonly studied in multifocal intraocular lenses [19]. Most studies relied on self-developed PRO instruments but did not provided any data on their validity or reliability. Only one study used a formally developed questionnaire called the Near Activity Visual Questionnaire (NAVQ) [3] for assessing vision clarity at close distances. This questionnaire was identified as the sole instrument designed to assess difficulties in near-vision function specifically in individuals with presbyopia. However, it has been suggested that a qualitative study is necessary to validate its content [20]. Moreover, this questionnaire does not encompass all the domains associated with the presbyopic condition [21], thereby limiting its ability to provide a comprehensive measure of the impact of presbyopia on patients. Several other tools are available for assessing MCL performance-related domains [3, 22–26], but none of them have been specifically developed for the target population. According to the COSMIN steering committee, “content validity is the most crucial measurement property of patient-reported outcome measures”. In order to achieve a strong content validity, it is essential to ensure the relevance, comprehensiveness, and comprehensibility of the item bank. One of the key factors in achieving this is consulting with the target population, as their input plays a vital role in ensuring the quality of the content [27].

The objective of this study is to develop an item-bank integrating all domains necessary to assess the patient’s perspective on multifocal contact lens performance, offering a comprehensive measure. The item-bank will ensure a high level of content validity, be self-administered, and will initially be available in Spanish (Additional file 1). The aim of this tool is to serve as a valuable resource for research and optometric clinics. The item-bank is expected to aid clinicians in understanding how to assess various aspects associated with adapting to multifocal contact lenses in their practice. Additionally, it should support researchers in crafting items within these domains, making it easier to monitor patients with presbyopia who either already wear multifocal contact lenses or are newly starting to use them.

## Methods

**Fig. 1:**
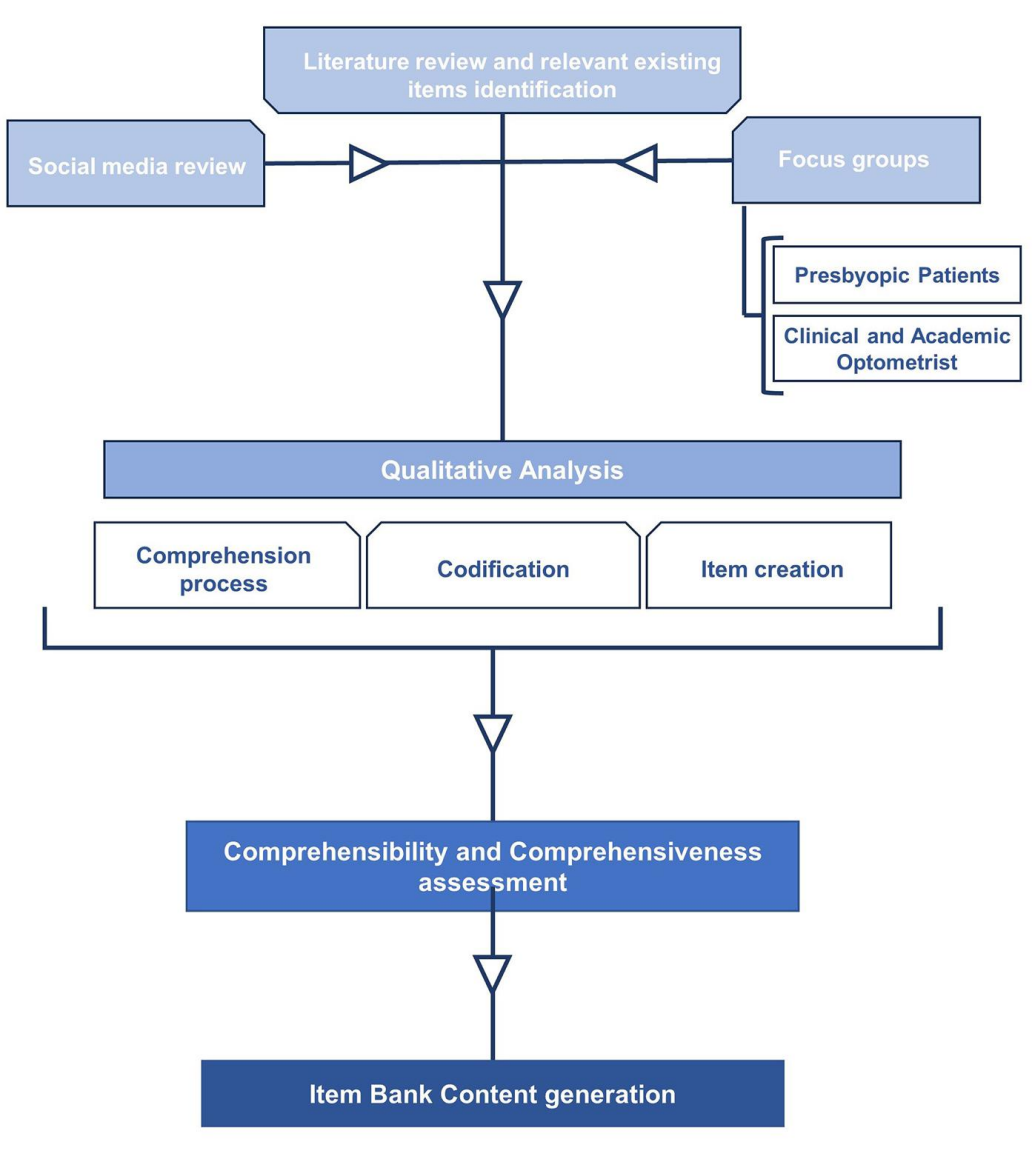
MCL-Pro instrument creation flow chart. Flow chart summarizes all the steps followed in the item bank creation. Each arrow indicates action leading to the box, presented sequentially

To establish the content of the MCL-PRO item bank, a systematic and stepwise inductive approach was undertaken, following the recommendations outlined in the COSMIN guidelines [27] and similar studies [21, 28]. The process involved the following steps (Fig. 1): (1) Literature review and relevant existing items identification (2) Social media review, (3) Semi-structured focus groups, (4) performing qualitative analysis, (5) refining and revising the items, and (6) generating the content of the item bank.

### Literature review and relevant existing items identification

The literature review exploration strategy employed an iterative approach without any specific time constraints, although it was concluded on December 1, 2021. The search was conducted in English on PubMed platform using the following (“PRO instrument” OR “patient reported outcomes” OR “patient-reported outcomes” OR “questionnaire” OR “item-bank”) AND (“refractive correction” OR “contact lens” OR “presbyopia”) AND (“development” OR “Validation”). Exclusion criteria was:

- Conference abstracts, case studies, case reports.
- Studies assessing satisfaction using non-standardised measures, scales or PRO instruments.
- Studies developing patient reported outcomes instrument without patient consultation during the content development phase.

These papers facilitated the identification of initial concepts and served as the basis to create a guide for conducting focus groups [28, 29].

Furthermore, all PRO instruments mentioned in the articles obtained from the search were selected for further analysis. The inclusive criteria for selection were as follows:

1. Instruments developed using valid content development methods, such as structured/semistructured interviews and/or literature reviews.
2. Instruments developed with target populaton consultation
3. Instruments evaluating MCL performance-related domains.

The chosen PRO instruments were employed to identify not only relevant and/or useful items but also other domains that could be associated with the topic.

### Social media review

A retrospective, non-interventional study was performed, analyzing social media data from the public platform twitter. The aim of the study was to investigate the impact of presbyopia, a condition related to aging eyes, and its various correction methods on individuals’ everyday lives. Furthermore, the study sought to examine how people commonly express this experiences through social media [30]. The search was conducted in Spanish using the web browser on May 10, 2022 and capture with google extension Ncapture for NVivo (version 1.0.290.0). The following terms were utilized: ‘presbicia’, ‘vista cansada’, ‘lentes progresivas’, ‘lentillas progresivas’ and ‘#presbicia’. The inclusion criteria was as follows:

1. Post in any official language from Spain.
2. Post with no commercial purposes.
3. Post from individuals.
4. Appropriate post. Not including any verbal violence to other members of the platform or swearing.

Consequently, any post sharing symptomatology or expressing concerns in any manner, including jokes, were selected for analysis.

### Focus groups

Between January 31, 2022, and May 11, 2022, a series of focus groups (FG) were carried out. These sessions had a duration of 30–45 min each and were online meetings that were recorded and verbatim transcribed. The meetings would follow a guide previously designed by the moderator [31]. The moderator (E.A.), a female optometrist from the research team, received training from a experienced researcher on this field and in conducting sessions of this nature (M.G). The moderator’s role extended beyond hosting the focus groups; they were also responsible for developing the focus group protocol and interview guide (Additional file 2). It should be noted that the guide was not strictly adhered to, as the primary objective was to establish a secure and open environment where participants could freely communicate with others who shared similar conditions. This allowed them to express their concerns, feelings, symptoms, experiences, and opinions. Therefore, each focus group consisted of 3–7 participants [29].

Prior to the focus group sessions, participants were provided with a participant information sheet and consent form. These documents had been approved by the ethics committee on January 12, 2022. Participants signed the consent form, indicating their agreement to participate in the focus group. This way, it was agreed that the focus group discussions would be audio recorded using Teams for later verbatim transcription and analysis.

The entire iterative process of collecting, analyzing, and refining data through interviews aims to achieve theoretical saturation. The concept of saturation is defined as the stage in the data collection process when no new conceptually relevant information emerges from individual interviews or focus groups, or when no additional information is deemed missing during cognitive interviewing. In this study, data saturation was evaluated following COSMIN guidelines [27]. To document this evaluation, a saturation table was created and updated throughout the qualitative analysis process. The table organized the information based on concepts derived from successive focus groups (FGs). The saturation stopping point was determined when the total sample size was more than 20 interviewees [32] and two consecutive focus groups revealed no new concepts [33].

In this study, two distinct focus groups were established: (1) Presbyopic patients’ focus groups (PFG) and (2) Clinical and Academic Optometrist focus groups (OFG). Saturation was independently evaluated for both types of FGs.

#### Presbyopic patients focus groups (PFG)

Via convenience sampling, participants were recruited from one of Alain Afflelou’s Optician stores (C. de Orense, 23, 28020 Madrid, Spain). And in order to enlarge the number of participants, snowball sampling was allowed. The Inclusion criteria was:

- Subjects aged over 40.
- MCL users or patients starting an MCL adaptation process.
- Established presbyopes or pre-presbyopes.
- Participants who did not utilize any form of correction or typically relied on single vision contact lenses or spectacles.
- Not presenting any eye disease.
- Not having any communication issues.
- Having Spanish as their native language.

Alain Afflelou introduced the participants to the research group and provided participants with a complimentary visual exam. In turn Mark’ennovy provided multifocal contact lens for trial. This allowed for multiple focus groups to be conducted with the same participants at various stages of the adaptation process. As a result, patients initially participated in a first focus group where the interview guide was followed. Subsequently, patients who were still undergoing the adaptation process or had started a new MCL trial took part in an additional focus group to share their experiences once their optometrist judged the adaptation to be complete.

#### Clinical and academic optometrist focus groups (OFG)

Optometrist with experience in MCL adaptations from Alain Afflelou volunteered to participate in the OFG, as well as academics from the Optometry and vision department of Complutense of Madrid University. This provided a reality check of how presbyopia is experienced from a commercial, theoretical and clinical perspective. In this case, only one session was accomplished per FG, following the interview guide previously designed.

### Qualitative analysis

Nvivo (version 1.7, released in March 2020) was the chosen software to procure the theme identification and codification [28]. An inductive approach was utilized to analyze the information [27]. Two researchers (E.A. and M.G.) independently and systematically identified themes, categories, or theories that surfaced from the data. This inductive analysis involved an iterative process of coding, categorizing, and analyzing the data to discover patterns and concepts. It provided an opportunity for exploring novel ideas and theories that might not have been initially anticipated before the study. Hence, to produce a conceptual map, define the domains and create of items from text quotations, three phases were accomplished: (1) a comprehension process, (2) codification and (3) item creation [34].

#### Comprehension process

In the comprehension process all information from bibliographic review, social media and FG verbatin transcriptions was read and preliminary observations were noted. This led to the identification of initial domains.

#### Codification

Codification was conducted as an independent and dynamic process. The domains identified in the literature review were utilized, and new domains could emerge or be integrated into existing ones if there was insufficient information to establish them as distinct categories. This comprehensive analysis led to the development of a conceptual map, capturing the synthesized information. In addition, the quotes supporting each domain were tallied and compared using chi-square analysis, followed by post-hoc analysis using the Bonferroni method. In line with other exploratory studies, statistical significance was attributed to P values below 0.05 [35].

#### Item creation

Both, the question format and the response options were created following the format proposed in validated items from other questionnaires, besides economic issues domain, as no useful evidence was found to form the responses. Hence, the items and responses in this domain were exclusively developed with the assistance of quotes extracted from focus groups. The literature reviewed suggests that the domains related to symptoms evaluation could be rated by 3 different scales, frequency, severity and bothersome [22]. The items included in the pool were designed with a 4 options verbal scale, as literature suggested that 4–6 was the optimal set of responses, however 4 have shown to be more simple to understand and be as precise as other more extensive scales [36]. The 5-point scale was discarded as a strategy to encourage presbyopes to express either a positive or negative opinion [37], avoiding possible future complications when optimizing scales and optimizing response rate [38, 39] In addition some cases there where items that could be answered referring on the current correcting method that the patient had; spectacles, CL or nothing [40]. Non-applicable option could be included if the patient did not wear the asked correction type or in items asking about certain activities or situations in order to give the res ponder the opportunity to express if an activity had not been done or they had not been exposed to a certain situation (Additional file 3).

### Comprehensibility and comprehensiveness assessment

Initially, the item bank was administered to the target population as part of a pilot test. The pilot test was designed for participants to respond to the 604 items over the course of 8 days. Each day, participants will receive 74–76 questions to complete. This decision was made to reduce the workload of the volunteers. The main objective of the pilot test was to assess the comprehensibility and comprehensiveness of the items [27]. Patients reported that some of the questions were unsuitable, as they appeared repetitive, overly lengthy, or difficult to comprehend. Consequently, a revision of the items was performed. The revision process involved the collaboration of three researchers: the director of the Optometry and Vision Department at the Optometry Faculty of Complutense University of Madrid (A.L.); an experienced researcher and professor from the Optometry and Vision Department at Complutense University of Madrid (M.G.); and a PhD student at Complutense University of Madrid (E.A). An evaluation guide (Additional file 4), created by Mariano González-Pérez, following Streiner y Norman recommendations [41], was used. This guide evaluated:

1. Wording clarity: items in the questionnaire should be easily understandable for the general public.
2. Length:items should be as concise as possible without sacrificing comprehensibility.
3. Ambiguity: items should aim to minimize multiple possible interpretations, as they can lead to uncertainty or confusion. Ambiguity can arise from various factors, including vague language, multiple meanings of words, unclear grammar, or lack of context. Additionally, when combined with an imposed response scheme, ambiguity can potentially force the subject to provide incorrect responses.
4. Double questions: items that present two inquiries simultaneously, each of which could receive distinct answers.
5. Jargon: terms associated with the jargon can unintentionally appear when writing an item, making them difficult for participants to understand.
6. Suggestive questions: the inclusion of specific words in an item can lead the interviewees to respond in a particular manner, thereby influencing their answers.
7. Personal impression: the evaluators’ perception of this item and its appropriateness for inclusion in the questionnaire.

For each evaluation category previously mentioned, the evaluators had to assign a score ranging from 1 to 5, with 1 being the lowest and 5 being the highest, for each item. The average score given by the evaluators was calculated. Top-rated items served as a guide to rewrite the lowest-rated. To ensure that the set of items was reduced to a more representative set and that items were correctly grouped under the correct domain, winnowing and binning was applied [27, 28]. The item culling criteria used was: item inconsistent with the domain definition, item similar in meaning with other items, item content too narrow to have wider applicability, and item confusing or unclear [34].

## Results

### Literature review and relevant existing items identification

A total of 52 articles were initially identified through the search, out of which 15 were determined to be relevant. Further analysis led to the removal of 3 articles due to lack of patient consultation in their content generation or a number of interviews less than 20. Among the remaining 12 articles, 9 included focus group consultation in their methodology [3, 22, 26, 42–45], 1 utilized personal interviews [46], and 2 were literature reviews [20, 47]. Additionally, 13 questionnaires with related domains were analyzed in order to identify useful validated items [3, 22–26, 40, 43, 45, 48–51].

### Social media review

Initially, a total of 226 tweets were identified through the search, out of which 127 were found to have no commercial purposes. Upon further analysis, it was revealed that only 81 tweets contained relevant content for our specific purpose.

### Focus groups

In this research, 14 focus groups, with a total of 54 participants, were conducted. The patient FGs had 24 participants, while the clinician FGs had 30 participants. Saturation was reach in both cases and documented (Tables 1 and 2).

**Table 1:** Clinical and Academic Optometrist FGs domain saturation table. Appreciate that all domains where completed after 2–3 focus group. Except from general symptoms which in spite of the moderator bringing the subject to the discussion no optometrist seemed to bring any new information up. In addition it is important to note that cognitive issues didn’t have two consecutive focus group before reaching saturation

Clinical and academic optometrist FGs	A	B	C	D	E
Cognitive issues		X		X	
Economic issues	X	X	X		
Emotional well-being	X	X	X		
Convenience	X	X	X		
Ocular symptoms	X	X	X		
General symptoms					
Visual symptoms	X	X			
Activity limitation	X		X

**Table 2:** Presbyopic Patients FGs domain saturation table. In the focus groups, before starting the adaptation process to a new contact lens, all domains reached the saturation pint after the 3rd focus group

Presbyopic patients FGs	A	B	C	D	E
Cognitive issues	X	X			
Economic issues	X	X			
Emotional well-being	X	X	X		
Convenience	X	X	X		
Ocular symptoms	X	X			
General symptoms	X		X		
Visual symptoms	X	X	X		
Activity limitation	X	X			

On the one hand, a total of 9 presbyopic patients FGs, each one with 3–7 participants were accomplished. On the other hand, 5 clinicians FGs were needed to reach saturation, each of them composed from 5 to 7 participants, a total of 23 Clinical Optometrist and 7 academic optometrist.

#### Presbyopic patients focus groups

Patient focus groups were composed of 14 women and 10 men, between 46 and 58 years old ($52 \pm 5$). Volunteers could present different refractive errors in addition to presbyopia. Among them, 7 were emmetropic, 10 had myopia, 7 had hypermetropia, and 5 presented astigmatism.

#### Clinical and academic optometrist focus groups

Clinical and Academic Optometrist focus groups were composed of 21 women and 8 men, of $41 \pm 4$ years old. The media of worked years on the optometric sector was $19 \pm 9$ and the number of MCL prescribed per month $9 \pm 4$.

### Qualitative analysis

**Fig. 2:**
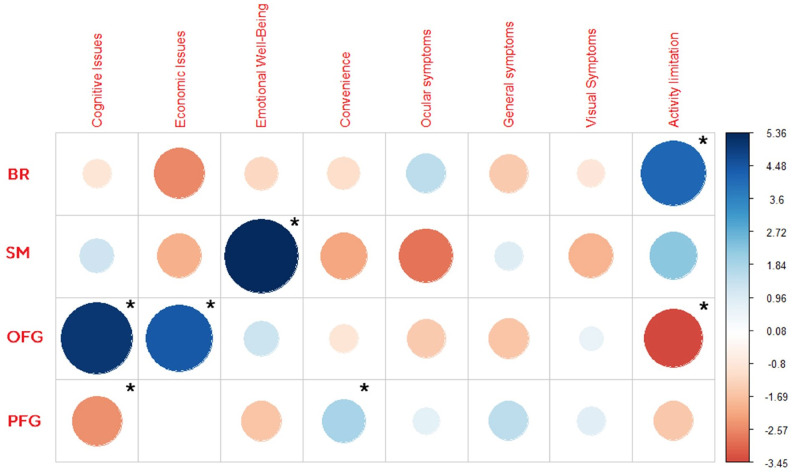
Chi-square and post-hoc analysis. The expected number of observations is compared to the recorded number of observations. The magnitude of this difference (residuals) is indicated in the right column. Fields marked with “*” indicate a significant value

The creation of domains and items was achieved through the analysis of 1.339 quotes that were found to be relevant. Originally, 304 references were from literature review and existing relevant PROs; from social media review, 127 had no commercial purposes from which 81 were relevant; and 954 were detected through focus group discussion (Table 3). Significant differences in the number of references obtained for each domain were observed based on the source (BR, SM, OFG, PFG) as indicated by the Chi-square test. Additionally, the subsequent Post-hoc analysis confirmed that the differences were statistically significant (Fig. 2).

**Table 3:** Number of references per domain. Bibliographic and PRO instruments review (BR) contributed with a total of 304 references; Social media (SM) added 81 references, in clinical and Academic Optometrist FGs (OFG) 203 references where found and patients focus groups (PFG) contributed with 751 references

	BR	SM	OFG	PFG	Total per domain
Cognitive issues	8 (0.6%)	5 (0.4%)	21 (1.6%)	14 (1%)	48 (3.6%)
Economic issues	5 (0.4%)	0	24 (1.8%)	37 (2.8%)	66 (5%)
Emotional well-being	36 (2.7%)	30 (2.2%)	36 (2.7%)	91 (6.8%)	193 (14.4%)
Convenience	82 (6,1%)	14 (1%)	55 (4,1%)	257 (19.2%)	408 (30.5%)
Ocular symptoms	40 (3%)	0	14 (1%)	84 (6.3%)	138 (10.3%)
General symptoms	1 (0.1%)	2 (0.1%)	0	15 (1.1%)	18 (1.3%)
Visual symptoms	43 (3.2%)	6 (0.4%)	36 (2.7%)	130 (9.7%)	215 (16%)
Activity limitation	89 (6.6%)	24 (1.8%)	17 (1.3%)	123 (9.2%)	253 (18.9%)
Total per origin	304 (22.7%)	81 (6%)	203 (15.2%)	751 (56.1%)	1339 (100%)

#### Domain and item generation

**Fig. 3:**
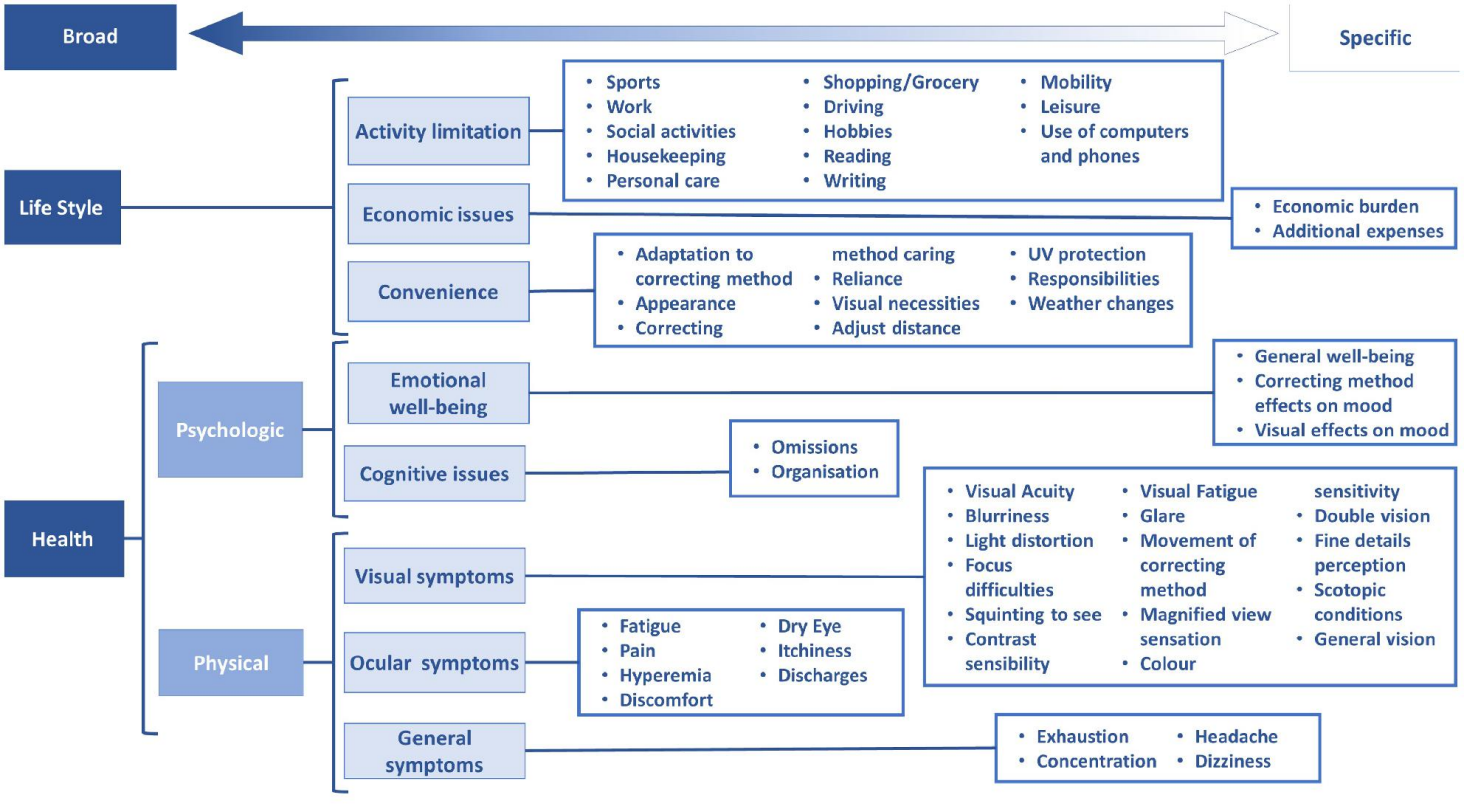
MCL-Pro content conceptual map. Conceptual map of MCL-Pro instrument content placing the health and life style domains included in a theoretical context, presented sequentially

Initially, thought the comprehension process a total of 9 possible domains were found. However after a further content analysis, references were condense under the different codes, from specific to more broad concepts (Fig. 3), and one of the domains named self-confidence was absorbed by emotional well-being. This led to the construction of 8 well defined domains, important for patient’s MCL performance perspective, that would host the items; cognitive issues, economic issues, emotional well-being, convenience, ocular symptoms, general symptoms, visual symptoms and activity limitation (Table 4). Names of the official domains and definitions are based on bibliographic review and other questionnaires.

**Table 4:** Domain name and definition. This definition was followed while ordering references and items

Domain	Definition
Cognitive issues	Problems arising from a persons’ difficulty to think, learn, remember or make decisions.
Economic issues	Economic implication for presbyopia correction selection.
Emotional well-being	Emotional and/or psychological problems that an individual might face or that are considered to be related to presbyopia.
Convenience	The quality of an individual’s comfort, time consumption, needs, desires and purposes that are compromised due to the presbyopic condition.
Ocular symptoms	Non-visual eye sensations related to ageing and presbyopia.
General symptoms	Unwanted non-ocular sensations.
Visual symptoms	Unwanted visual sensations due to the presbyopic condition or correction.
Activity limitation	Difficulties that a person might face when performing specific activities due to the presbyopic condition.

**Fig. 4:**
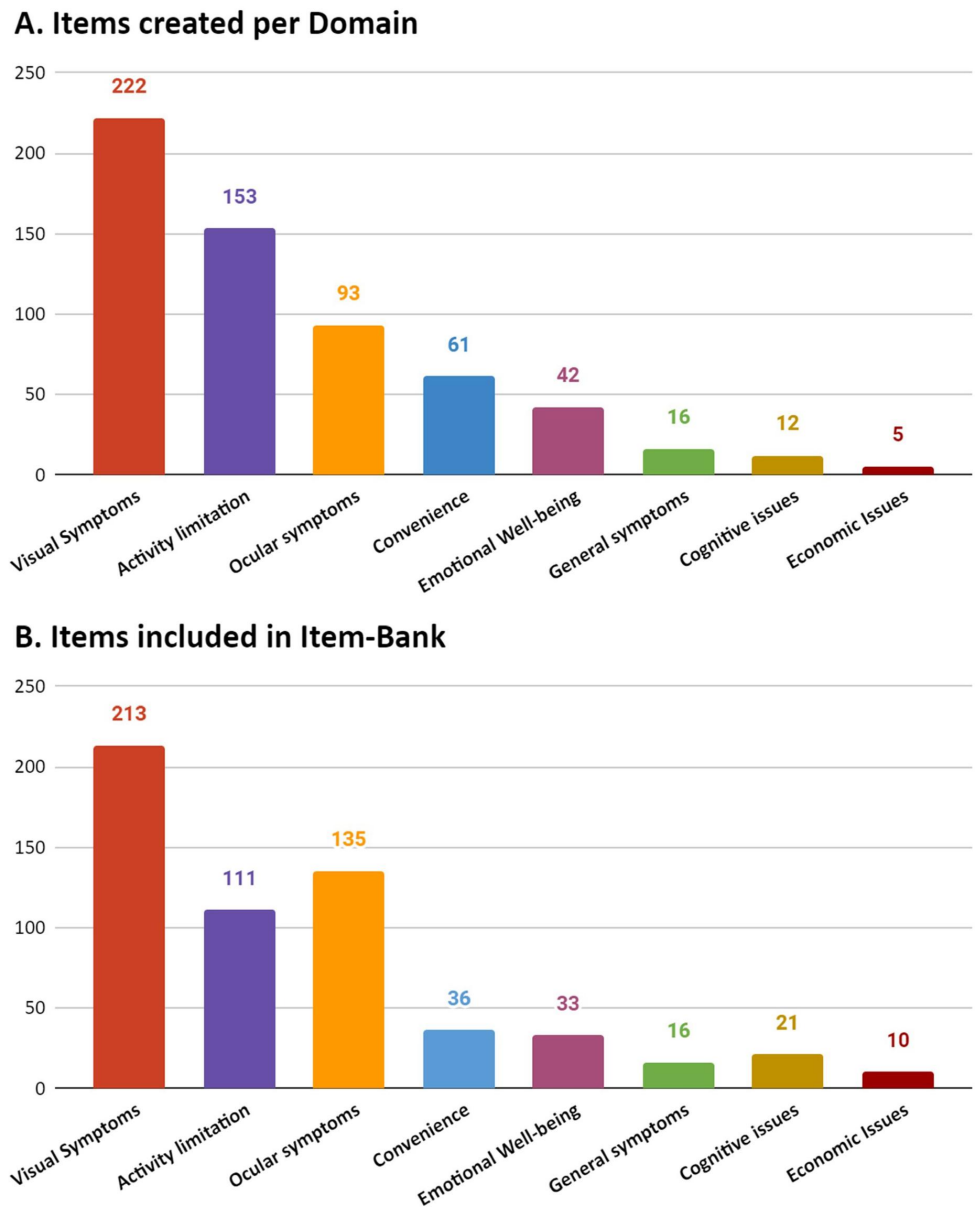
(**A**) Items created per domain. (**B**) Items included in the Item-Bank. (**A**) The graph shows the number of items initially created per domain (**B**) Items included after comprehensibility and comprehensiveness assessment

Subsequently, 604 items were initially created (Additional file 3; Fig. 4A). Items from Activity limitation, general, visual and ocular symptoms where asked for 3 possible correcting methods.

#### Comprehensibility and comprehensiveness assessment

Initially, the items were distributed to 31 patients for feedback on relevance, comprehensiveness, and comprehensibility. However, only 13 patients agreed to participate in the analysis. These participants mentioned that the items were repetitive, lengthy, and difficult to understand. As a result, it was decided to conduct a more comprehensive analysis of content validity, following a specific guide. After the item revision accomplished following the evaluation guide. A total of 575 items where selected to form part of the item-bank (Fig. 4B). It is notable that domains such as ocular symptoms or economic issues experimented a growth in terms of items, while others such as activity limitation had several items deleted.

## Discussion

### Data collecting method

In this study three different data collecting methods were used. Traditional methods such as the focus groups and the literature review and a less common one, the social media review.

Social media review, trough twitter primarily produced brief quotes that often lacked sufficient detail and context. This limitation may have been influenced by character constraints on this social media platform. Without the opportunity to probe topics further, some quotes were vague and ambiguous, diminishing their clarity and interpretability. Furthermore, ensuring that the sample comprised individuals with confirmed diagnoses of presbyopia is not possible, as noted in a similar study [21].

The literature often provided limited summaries of clinical characteristics, resulting in restricted generalizability of the information. Although these two methods may differ and provide less in-depth information compared to focus groups, it was found to be extremely beneficial for the moderator to have familiarity with the general topics discussed through them. This familiarity aided in the development of a guide for the focus groups [28, 29, 31].

As expected, the focus groups provided a more thorough exploration of concepts. This can be attributed to the dynamic interaction between the moderator and the diverse group of participants, allowing for a deeper discussion of topics and the connection of concepts based on the various experiences shared. In this group setting, one person’s opinion or story would often trigger the recall of additional information from other participants, which might have been overlooked in individual interviews. It is worth emphasizing that conducting multiple focus groups with presbyopic patients can create a more comfortable environment for them. This extended interaction allows patients to develop trust with the moderator over time, enabling them to share their perspectives in a more natural and authentic manner. However, focus groups do come with their own set of limitations. Participants may tend to provide socially desirable responses, leading to a consensus rather than the generation of novel ideas. Furthermore, the presence of others in the group can potentially influence an individual’s self-perception and their perception of the topic under discussion. Additionally, going into great depth with the subjects may prove challenging [52].

#### Saturation point

According to the COSMIN guidelines [27], the initial step in assessing the quality of PROM development involves consulting the target population. In this study, we not only incorporated the perspectives of patients but also included input from clinicians. To ensure data saturation, saturation grids were utilized, providing evidence of data collection until saturation was achieved. The literature identified two standardized rules for achieving saturation, both of which were successfully applied in this study for seven out of eight domains. The only exception was observed in the focus groups consisting of clinical and academic optometrists, specifically regarding cognitive issues. As shown in Table 2, no two consecutive focus groups in this domain yielded new information. However, according to the literature “the point in the data collection process when no new concept-relevant information is being elicited from individual interviews or focus groups, or no new information is deemed missing during cognitive interviewing” [32]. Meaning that we should not only rely on the saturation tables to asses content saturation [53]. Based on the analysis, it was observed that clinicians in the focus group provided minimal information regarding the cognitive issue domain. Among the clinical optometrist focus groups, Focus Group D emerged as the most informative and also the largest in terms of participants. Considering that only 2 out of the 5 focus groups contributed information, it was assumed that saturation had been achieved and that it adequately represented all aspects of the measurement concept from the perspective of the patient population of interest.

Furthermore, no new information regarding general symptoms was obtained from these groups. Clinicians did not appear to associate these symptoms with the success of MCL fitting or performance. This highlights a potential communication gap between patients and clinicians, as it has been previously suggested [17].

### Qualitative analysis

The FGs (Focus Groups) research method has been found to generate a significant amount of content. However, when examining Table 4, it becomes evident that bibliographic review produces more citations compared to OFGs alone. This difference could be attributed to the fact that all the reviewed articles and questionnaires incorporated patient consultation, which emerged as the most informative method, contributing to the total with 56% of the quotes.

On the other hand, SM emerged as the method contributing the fewest quotes, amounting to only 6%. In a similar study [21], a significantly larger quantity of quotes was discovered and analyzed. This disparity may be attributed to the study being conducted across multiple platforms, including Twitter, forums, blogs, and news posts. However, it is important to acknowledge that the researchers also found that the information obtained through SM was not as valuable as other methods. Furthermore, they argued that the presbyopic condition is not perceived as sensitive by individuals, leading to more open discussions in interviews, unlike studies involving more delicate conditions where SM proved to be more useful. In spite of that SM showed a significant number of quotes in the domain of emotional well-being. One possible reason for this could be that individuals may not feel as comfortable expressing their feelings in focus groups, where they share information with unfamiliar individuals. This notion is supported by the fact that clinicians, a much more familiar person to our target population, also indicate the importance of emotional well-being when assessing MCL satisfaction. Clinicians particularly emphasized the significance of motivation and self-contentment. Additionally, it is worth noting that only three questionnaires [26, 40, 44] included items specifically addressing emotional well-being. This highlights the need to utilize alternative methods to further explore and elaborate on certain domains, even though patient consultation and bibliographic review proved to be the most informative methods.

When analyzing the different domains, convenience stands out as the most extensively discussed topic, accounting for 30% of the quotes. Notably, convenience consistently ranked among the top three popular topics across all research methods, but specially in PFG. Activity limitation and Visual symptoms also emerged as prominent domains in three out of the four methods. However, significant differences were observed between the methods. However Activity limitation had significant minimal contribution in OFGs (Fig. 2), but was extensively discussed in SM. Conversely, Visual symptoms received more attention in OFGs compared to SM. In contrast, the domains of general symptoms and cognitive issues, followed by economic issues, had the fewest number of citations. However, it is important to emphasize that despite their lower frequency, these domains are still significant. This is supported by the statistical results regarding cognitive issues, which demonstrated their significant relevance in both PFG and OFG (see Fig. 2). It is worth noting that cognitive issues are not typically addressed in similar questionnaires, further highlighting their importance in this study [3, 26]. In addition, although there were only a few specific aspects that bothered patients in terms of general symptoms (Fig. 2), these concerns held significance for them. This perspective was not necessarily reflected by clinicians, as previously mentioned. However, clinicians did consider economic issues to be a more significant domain, which, in proportion, was not as well reflected in the other research methods.

### Final item-bank

The items included in the MCL item bank represent a comprehensive representation of the conceptual model developed in this study. The significance of a thorough review process following item creation is evident. The COSMIN guidelines [27] recommend evaluating content quality through consultation with both patients and professionals. Unfortunately, patient consultation was not feasible in this study. Nevertheless, alternative methods have been employed in other studies to assess content quality, such as expert panel sessions of binning and winnowing [34] or following an item assessment guide [51].

The majority of items in the item bank are categorized under domains associated with symptoms. The variation in the quantity of items within these domains is primarily due to the utilization of three different scales to evaluate a single symptom. Upon overall examination, it is evident that the most prevalent domains are Visual symptoms, followed by ocular symptoms, activity limitation, and convenience. These findings align with the results obtained from the qualitative analysis of bibliographic sources.

However, it is worth noting that this item bank also includes a substantial number of items addressing psychological aspects (Fig. 2) such as emotional well-being and cognitive issues. These domains are not extensively represented in many questionnaires. Additionally, the introduction of the concept of economic issues within the lifestyle category is noteworthy. Previously, this aspect had only been addressed by three items in the CLIQ [26] and two items in the QIRC [44] questionnaires.

### Study limitations

The limitations faced in this study are mainly:

1. The limited social media platforms consulted in the SM research. Other studies explored blogs and news posts, yielding a significantly greater number of quotes. However, in this study, the number of quotes identified was so extensive that 50% of them had to be excluded from the analysis. Despite this, their findings regarding the quality of the information found did not differ from ours [21].
2. The lack of patient consultation on the items created. In this study it was not possible due to the difficulties face when finding volunteers. However, as it has been previously mentioned, other methods were employed in to assess the content quality [47, 51].
3. The absence of the evaluation of a valid scoring system for the generated items. Limiting the use of the items but presenting a potential topic for future research.

Therefore, there is no reason to believe that the limitations encountered in this study have a significant impact on the findings obtained.

## Conclusion

An extensive item bank has been meticulously developed using various standardized methods, aiming to englove all the relevant aspects for evaluating MCL performance from the patient’s perspective.

### Electronic supplementary material

Below is the link to the electronic supplementary material.

**Additional File 1: Item-bank in Spanish.** This document contains all the questions included in the item-bank

**Additional File 2: Focus Group Guide.** This document contains the guide that the moderator followed during the focus groups. The content is in Spanish since the focus groups were conducted in this language

**Additional File 3: Item construction information.** This file is compose of 2 tables. This material did not undergo formal translation from Spanish to English, therefore a cross-cultural validation process should be performed before it is used. Table 1: presents examples of items included in the item bank. Table 2: shows quotes extracted from the different research methods supporting an item. This table should be read horizontally

**Additional File 4: Item review guide.** In this document you can read the guide followed for comprehensibility and comprehensiveness assessment. The content is in Spanish since the items are written in this language and it is the first language of the evaluators

## Data Availability

Data and materials will be available upon request.
