# Supplementary material for: Content development for a new item-bank for measuring multifocal contact lens performance
Source: J Patient Rep Outcomes. 2024 Feb 8;8:16. doi: 10.1186/s41687-024-00689-w (PMC10853121; doi:10.1186/s41687-024-00689-w)
Supplement: Supplementary file 1 — Additional File 1: Item-bank in Spanish. This document contains all the questions included in the item-bank [file 41687_2024_689_MOESM1_ESM.pdf]

Multifocal contact lensperformance Item-bank

id	trait	Mientras usa gafas ¿Cómo de cierta es la siguiente afirmación para usted?	
11001	ActivityLimitation	Debido a mi visión, me resulta difícil salir a dar un paseo.	a. Totalmente falsa.<br>b. Bastante falsa.<br>c. Bastante cierto.<br>d. Totalmente cierto.<br>e. No he hecho esta actividad.
11002	ActivityLimitation	Me cuesta afeitarme.	a. Totalmente falsa.<br>b. Bastante falsa.<br>c. Bastante cierto.<br>d. Totalmente cierto.<br>e. No he hecho esta actividad.
11003	ActivityLimitation	Me cuesta aparcar el coche.	a. Totalmente falsa.<br>b. Bastante falsa.<br>c. Bastante cierto.<br>d. Totalmente cierto.<br>e. No he hecho esta actividad.
11004	ActivityLimitation	Me cuesta comer o cenar con otras personas.	a. Totalmente falsa.<br>b. Bastante falsa.<br>c. Bastante cierto.<br>d. Totalmente cierto.<br>e. No he hecho esta actividad.
11005	ActivityLimitation	Me cuesta conducir de día.	a. Totalmente falsa.<br>b. Bastante falsa.<br>c. Bastante cierto.<br>d. Totalmente cierto.<br>e. No he hecho esta actividad.
11006	ActivityLimitation	Me cuesta conducir de noche.	a. Totalmente falsa.<br>b. Bastante falsa.<br>c. Bastante cierto.<br>d. Totalmente cierto.<br>e. No he hecho esta actividad.
11007	ActivityLimitation	Me cuesta conducir mientras llueve.	a. Totalmente falsa.<br>b. Bastante falsa.<br>c. Bastante cierto.<br>d. Totalmente cierto.<br>e. No he hecho esta actividad.

11009	ActivityLimitation	Me cuesta distinguir letra impresa. Por ejemplo, la carta de un restaurante.	a. Totalmente falsa.<br>b. Bastante falsa.<br>c. Bastante cierto.<br>d. Totalmente cierto.<br>e. No he hecho esta actividad.
11010	ActivityLimitation	Me cuesta escribir a mano.	a. Totalmente falsa.<br>b. Bastante falsa.<br>c. Bastante cierto.<br>d. Totalmente cierto.<br>e. No he hecho esta actividad.
11011	ActivityLimitation	Me cuesta hacer deporte.	a. Totalmente falsa.<br>b. Bastante falsa.<br>c. Bastante cierto.<br>d. Totalmente cierto.<br>e. No he hecho esta actividad.
11012	ActivityLimitation	Me cuesta hacer manualidades, como coser, pintar o bricolaje.	a. Totalmente falsa.<br>b. Bastante falsa.<br>c. Bastante cierto.<br>d. Totalmente cierto.<br>e. No he hecho esta actividad.
11013	ActivityLimitation	Me cuesta hacer mi trabajo.	a. Totalmente falsa.<br>b. Bastante falsa.<br>c. Bastante cierto.<br>d. Totalmente cierto.<br>e. No he hecho esta actividad.
11014	ActivityLimitation	Me cuesta hacer tareas cotidianas, como limpiar, hacer la compra o cocinar.	a. Totalmente falsa.<br>b. Bastante falsa.<br>c. Bastante cierto.<br>d. Totalmente cierto.<br>e. No he hecho esta actividad.
11015	ActivityLimitation	Me cuesta hacerme cargo de niños.	a. Totalmente falsa.<br>b. Bastante falsa.<br>c. Bastante cierto.<br>d. Totalmente cierto.<br>e. No he hecho esta actividad.
11016	ActivityLimitation	Me cuesta leer algo que yo mismo he escrito.	a. Totalmente falsa.<br>b. Bastante falsa.<br>c. Bastante cierto.<br>d. Totalmente cierto.<br>e. No he hecho esta actividad.

11017	ActivityLimitation	Me cuesta leer las etiquetas de la ropa.	a. Totalmente falsa.<br>b. Bastante falsa.<br>c. Bastante cierto.<br>d. Totalmente cierto.<br>e. No he hecho esta actividad.
11018	ActivityLimitation	Me cuesta leer letra pequeña, como el prospecto de un medicamento.	a. Totalmente falsa.<br>b. Bastante falsa.<br>c. Bastante cierto.<br>d. Totalmente cierto.<br>e. No he hecho esta actividad.
11019	ActivityLimitation	Me cuesta leer letreros de la calle, como indicaciones o nombres de calles.	a. Totalmente falsa.<br>b. Bastante falsa.<br>c. Bastante cierto.<br>d. Totalmente cierto.<br>e. No he hecho esta actividad.
11020	ActivityLimitation	Me cuesta leer letreros luminosos en la calle.	a. Totalmente falsa.<br>b. Bastante falsa.<br>c. Bastante cierto.<br>d. Totalmente cierto.<br>e. No he hecho esta actividad.
11021	ActivityLimitation	Me cuesta leer un libro.	a. Totalmente falsa.<br>b. Bastante falsa.<br>c. Bastante cierto.<br>d. Totalmente cierto.<br>e. No he hecho esta actividad.
11022	ActivityLimitation	Me cuesta maquillarme.	a. Totalmente falsa.<br>b. Bastante falsa.<br>c. Bastante cierto.<br>d. Totalmente cierto.<br>e. No he hecho esta actividad.
11023	ActivityLimitation	Me cuesta nadar.	a. Totalmente falsa.<br>b. Bastante falsa.<br>c. Bastante cierto.<br>d. Totalmente cierto.<br>e. No he hecho esta actividad.
11024	ActivityLimitation	Me cuesta peinarme.	a. Totalmente falsa.<br>b. Bastante falsa.<br>c. Bastante cierto.<br>d. Totalmente cierto.<br>e. No he hecho esta actividad.

11025	ActivityLimitation	Me cuesta reconocer la cara de personas conocidas.	a. Totalmente falsa.<br>b. Bastante falsa.<br>c. Bastante cierto.<br>d. Totalmente cierto.<br>e. No he hecho esta actividad.
11026	ActivityLimitation	Me cuesta seguir una reunión de trabajo.	a. Totalmente falsa.<br>b. Bastante falsa.<br>c. Bastante cierto.<br>d. Totalmente cierto.<br>e. No he hecho esta actividad.
11027	ActivityLimitation	Me cuesta subir o bajar escaleras.	a. Totalmente falsa.<br>b. Bastante falsa.<br>c. Bastante cierto.<br>d. Totalmente cierto.<br>e. No he hecho esta actividad.
11028	ActivityLimitation	Me cuesta usar el móvil.	a. Totalmente falsa.<br>b. Bastante falsa.<br>c. Bastante cierto.<br>d. Totalmente cierto.<br>e. No he hecho esta actividad.
11029	ActivityLimitation	Me cuesta usar el teléfono fijo.	a. Totalmente falsa.<br>b. Bastante falsa.<br>c. Bastante cierto.<br>d. Totalmente cierto.<br>e. No he hecho esta actividad.
11030	ActivityLimitation	Me cuesta ver el ordenador.	a. Totalmente falsa.<br>b. Bastante falsa.<br>c. Bastante cierto.<br>d. Totalmente cierto.<br>e. No he hecho esta actividad.
11031	ActivityLimitation	Me cuesta ver la hora en un reloj de pared.	a. Totalmente falsa.<br>b. Bastante falsa.<br>c. Bastante cierto.<br>d. Totalmente cierto.<br>e. No he hecho esta actividad.
11032	ActivityLimitation	Me cuesta ver la hora en un reloj de pulsera.	a. Totalmente falsa.<br>b. Bastante falsa.<br>c. Bastante cierto.<br>d. Totalmente cierto.<br>e. No he hecho esta actividad.

11033	ActivityLimitation	Me cuesta ver la televisión.	a. Totalmente falsa.<br>b. Bastante falsa.<br>c. Bastante cierto.<br>d. Totalmente cierto.<br>e. No he hecho esta actividad.
11034	ActivityLimitation	Me cuesta ver una obra de teatro o una película en el cine.	a. Totalmente falsa.<br>b. Bastante falsa.<br>c. Bastante cierto.<br>d. Totalmente cierto.<br>e. No he hecho esta actividad.
11035	ActivityLimitation	Me cuesta viajar en avión.	a. Totalmente falsa.<br>b. Bastante falsa.<br>c. Bastante cierto.<br>d. Totalmente cierto.<br>e. No he hecho esta actividad.
11036	ActivityLimitation	Me cuesta viajar en transporte público.	a. Totalmente falsa.<br>b. Bastante falsa.<br>c. Bastante cierto.<br>d. Totalmente cierto.<br>e. No he hecho esta actividad.
11037	ActivityLimitation	Mientras hablo con alguien, me cuesta ver sus reacciones.	a. Totalmente falsa.<br>b. Bastante falsa.<br>c. Bastante cierto.<br>d. Totalmente cierto.<br>e. No he hecho esta actividad.
11038	ActivityLimitation	Me cuesta leer prospectos	a. Totalmente falsa.<br>b. Bastante falsa.<br>c. Bastante cierto.<br>d. Totalmente cierto.<br>e. No he hecho esta actividad.

id	trait	Mientras usa Lentillas ¿Cómo de cierta es la siguiente afirmación para usted?	
13001	ActivityLimitation	Debido a mi visión, me resulta difícil salir a dar un paseo.	a. Totalmente falsa.<br>b. Bastante falsa.<br>c. Bastante cierto.<br>d. Totalmente cierto.<br>e. No he hecho esta actividad.

13002	ActivityLimitation	Me cuesta afeitarme.	a. Totalmente falsa.<br>b. Bastante falsa.<br>c. Bastante cierto.<br>d. Totalmente cierto.<br>e. No he hecho esta actividad.
13003	ActivityLimitation	Me cuesta aparcar el coche.	a. Totalmente falsa.<br>b. Bastante falsa.<br>c. Bastante cierto.<br>d. Totalmente cierto.<br>e. No he hecho esta actividad.
13004	ActivityLimitation	Me cuesta comer o cenar con otras personas.	a. Totalmente falsa.<br>b. Bastante falsa.<br>c. Bastante cierto.<br>d. Totalmente cierto.<br>e. No he hecho esta actividad.
13005	ActivityLimitation	Me cuesta conducir de día.	a. Totalmente falsa.<br>b. Bastante falsa.<br>c. Bastante cierto.<br>d. Totalmente cierto.<br>e. No he hecho esta actividad.
13006	ActivityLimitation	Me cuesta conducir de noche.	a. Totalmente falsa.<br>b. Bastante falsa.<br>c. Bastante cierto.<br>d. Totalmente cierto.<br>e. No he hecho esta actividad.
13007	ActivityLimitation	Me cuesta conducir mientras llueve.	a. Totalmente falsa.<br>b. Bastante falsa.<br>c. Bastante cierto.<br>d. Totalmente cierto.<br>e. No he hecho esta actividad.
13008	ActivityLimitation	Me cuesta distinguir la hora en un reloj de pared.	a. Totalmente falsa.<br>b. Bastante falsa.<br>c. Bastante cierto.<br>d. Totalmente cierto.<br>e. No he hecho esta actividad.
13009	ActivityLimitation	Me cuesta distinguir letra impresa. Por ejemplo, la carta de un restaurante.	a. Totalmente falsa.<br>b. Bastante falsa.<br>c. Bastante cierto.<br>d. Totalmente cierto.<br>e. No he hecho esta actividad.

13010	ActivityLimitation	Me cuesta escribir a mano.	a. Totalmente falsa.<br>b. Bastante falsa.<br>c. Bastante cierto.<br>d. Totalmente cierto.<br>e. No he hecho esta actividad.
13011	ActivityLimitation	Me cuesta hacer deporte.	a. Totalmente falsa.<br>b. Bastante falsa.<br>c. Bastante cierto.<br>d. Totalmente cierto.<br>e. No he hecho esta actividad.
13012	ActivityLimitation	Me cuesta hacer manualidades, como coser, pintar o bricolaje.	a. Totalmente falsa.<br>b. Bastante falsa.<br>c. Bastante cierto.<br>d. Totalmente cierto.<br>e. No he hecho esta actividad.
13013	ActivityLimitation	Me cuesta hacer mi trabajo.	a. Totalmente falsa.<br>b. Bastante falsa.<br>c. Bastante cierto.<br>d. Totalmente cierto.<br>e. No he hecho esta actividad.
13014	ActivityLimitation	Me cuesta hacer tareas cotidianas, como limpiar, hacer la compra o cocinar.	a. Totalmente falsa.<br>b. Bastante falsa.<br>c. Bastante cierto.<br>d. Totalmente cierto.<br>e. No he hecho esta actividad.
13015	ActivityLimitation	Me cuesta hacerme cargo de niños.	a. Totalmente falsa.<br>b. Bastante falsa.<br>c. Bastante cierto.<br>d. Totalmente cierto.<br>e. No he hecho esta actividad.
13016	ActivityLimitation	Me cuesta leer algo que yo mismo he escrito.	a. Totalmente falsa.<br>b. Bastante falsa.<br>c. Bastante cierto.<br>d. Totalmente cierto.<br>e. No he hecho esta actividad.
13017	ActivityLimitation	Me cuesta leer las etiquetas de la ropa.	a. Totalmente falsa.<br>b. Bastante falsa.<br>c. Bastante cierto.<br>d. Totalmente cierto.<br>e. No he hecho esta actividad.

13018	ActivityLimitation	Me cuesta leer letra pequeña, como el prospecto de un medicamento.	a. Totalmente falsa.<br>b. Bastante falsa.<br>c. Bastante cierto.<br>d. Totalmente cierto.<br>e. No he hecho esta actividad.
13019	ActivityLimitation	Me cuesta leer letreros de la calle, como indicaciones o nombres de calles.	a. Totalmente falsa.<br>b. Bastante falsa.<br>c. Bastante cierto.<br>d. Totalmente cierto.<br>e. No he hecho esta actividad.
13020	ActivityLimitation	Me cuesta leer letreros luminosos en la calle.	a. Totalmente falsa.<br>b. Bastante falsa.<br>c. Bastante cierto.<br>d. Totalmente cierto.<br>e. No he hecho esta actividad.
13021	ActivityLimitation	Me cuesta leer un libro.	a. Totalmente falsa.<br>b. Bastante falsa.<br>c. Bastante cierto.<br>d. Totalmente cierto.<br>e. No he hecho esta actividad.
13022	ActivityLimitation	Me cuesta maquillarme.	a. Totalmente falsa.<br>b. Bastante falsa.<br>c. Bastante cierto.<br>d. Totalmente cierto.<br>e. No he hecho esta actividad.
13023	ActivityLimitation	Me cuesta nadar.	a. Totalmente falsa.<br>b. Bastante falsa.<br>c. Bastante cierto.<br>d. Totalmente cierto.<br>e. No he hecho esta actividad.
13024	ActivityLimitation	Me cuesta peinarme.	a. Totalmente falsa.<br>b. Bastante falsa.<br>c. Bastante cierto.<br>d. Totalmente cierto.<br>e. No he hecho esta actividad.
13025	ActivityLimitation	Me cuesta reconocer la cara de personas conocidas.	a. Totalmente falsa.<br>b. Bastante falsa.<br>c. Bastante cierto.<br>d. Totalmente cierto.<br>e. No he hecho esta actividad.

13026	ActivityLimitation	Me cuesta seguir una reunión de trabajo.	a. Totalmente falsa.<br>b. Bastante falsa.<br>c. Bastante cierto.<br>d. Totalmente cierto.<br>e. No he hecho esta actividad.
13027	ActivityLimitation	Me cuesta subir o bajar escaleras.	a. Totalmente falsa.<br>b. Bastante falsa.<br>c. Bastante cierto.<br>d. Totalmente cierto.<br>e. No he hecho esta actividad.
13028	ActivityLimitation	Me cuesta usar el móvil.	a. Totalmente falsa.<br>b. Bastante falsa.<br>c. Bastante cierto.<br>d. Totalmente cierto.<br>e. No he hecho esta actividad.
13029	ActivityLimitation	Me cuesta usar el teléfono fijo.	a. Totalmente falsa.<br>b. Bastante falsa.<br>c. Bastante cierto.<br>d. Totalmente cierto.<br>e. No he hecho esta actividad.
13030	ActivityLimitation	Me cuesta ver el ordenador.	a. Totalmente falsa.<br>b. Bastante falsa.<br>c. Bastante cierto.<br>d. Totalmente cierto.<br>e. No he hecho esta actividad.
13031	ActivityLimitation	Me cuesta ver la hora en un reloj de pared.	a. Totalmente falsa.<br>b. Bastante falsa.<br>c. Bastante cierto.<br>d. Totalmente cierto.<br>e. No he hecho esta actividad.
13032	ActivityLimitation	Me cuesta ver la hora en un reloj de pulsera.	a. Totalmente falsa.<br>b. Bastante falsa.<br>c. Bastante cierto.<br>d. Totalmente cierto.<br>e. No he hecho esta actividad.
13033	ActivityLimitation	Me cuesta ver la televisión.	a. Totalmente falsa.<br>b. Bastante falsa.<br>c. Bastante cierto.<br>d. Totalmente cierto.<br>e. No he hecho esta actividad.

13034	ActivityLimitation	Me cuesta ver una obra de teatro o una película en el cine.	a. Totalmente falsa.<br>b. Bastante falsa.<br>c. Bastante cierto.<br>d. Totalmente cierto.<br>e. No he hecho esta actividad.
13035	ActivityLimitation	Me cuesta viajar en avión.	a. Totalmente falsa.<br>b. Bastante falsa.<br>c. Bastante cierto.<br>d. Totalmente cierto.<br>e. No he hecho esta actividad.
13036	ActivityLimitation	Me cuesta viajar en transporte público.	a. Totalmente falsa.<br>b. Bastante falsa.<br>c. Bastante cierto.<br>d. Totalmente cierto.<br>e. No he hecho esta actividad.
13037	ActivityLimitation	Mientras hablo con alguien, me cuesta ver sus reacciones.	a. Totalmente falsa.<br>b. Bastante falsa.<br>c. Bastante cierto.<br>d. Totalmente cierto.<br>e. No he hecho esta actividad.
13038	ActivityLimitation	Me cuesta leer prospectos	a. Totalmente falsa.<br>b. Bastante falsa.<br>c. Bastante cierto.<br>d. Totalmente cierto.<br>e. No he hecho esta actividad.
id	trait	Sin usar gafas ni lentillas ¿Cómo de cierta es la siguiente afirmación para usted?	
12001	ActivityLimitation	Debido a mi visión, me resulta difí-cil salir a dar un paseo.	a. Totalmente falsa.<br>b. Bastante falsa.<br>c. Bastante cierto.<br>d. Totalmente cierto.<br>e. No he hecho esta actividad.
12002	ActivityLimitation	Me cuesta afeitarme.	a. Totalmente falsa.<br>b. Bastante falsa.<br>c. Bastante cierto.<br>d. Totalmente cierto.<br>e. No he hecho esta actividad.

12003	ActivityLimitation	Me cuesta aparcar el coche.	a. Totalmente falsa.<br>b. Bastante falsa.<br>c. Bastante cierto.<br>d. Totalmente cierto.<br>e. No he hecho esta actividad.
12004	ActivityLimitation	Me cuesta comer o cenar con otras personas.	a. Totalmente falsa.<br>b. Bastante falsa.<br>c. Bastante cierto.<br>d. Totalmente cierto.<br>e. No he hecho esta actividad.
12005	ActivityLimitation	Me cuesta conducir de día.	a. Totalmente falsa.<br>b. Bastante falsa.<br>c. Bastante cierto.<br>d. Totalmente cierto.<br>e. No he hecho esta actividad.
12006	ActivityLimitation	Me cuesta conducir de noche.	a. Totalmente falsa.<br>b. Bastante falsa.<br>c. Bastante cierto.<br>d. Totalmente cierto.<br>e. No he hecho esta actividad.
12007	ActivityLimitation	Me cuesta conducir mientras llueve.	a. Totalmente falsa.<br>b. Bastante falsa.<br>c. Bastante cierto.<br>d. Totalmente cierto.<br>e. No he hecho esta actividad.
12008	ActivityLimitation	Me cuesta distinguir letra impresa. Por ejemplo, la carta de un restaurante.	a. Totalmente falsa.<br>b. Bastante falsa.<br>c. Bastante cierto.<br>d. Totalmente cierto.<br>e. No he hecho esta actividad.
12009	ActivityLimitation	Me cuesta escribir a mano.	a. Totalmente falsa.<br>b. Bastante falsa.<br>c. Bastante cierto.<br>d. Totalmente cierto.<br>e. No he hecho esta actividad.
12010	ActivityLimitation	Me cuesta hacer deporte.	a. Totalmente falsa.<br>b. Bastante falsa.<br>c. Bastante cierto.<br>d. Totalmente cierto.<br>e. No he hecho esta actividad.

12011	ActivityLimitation	Me cuesta hacer manualidades, como coser, pintar o bricolaje.	a. Totalmente falsa.<br>b. Bastante falsa.<br>c. Bastante cierto.<br>d. Totalmente cierto.<br>e. No he hecho esta actividad.
12012	ActivityLimitation	Me cuesta hacer mi trabajo.	a. Totalmente falsa.<br>b. Bastante falsa.<br>c. Bastante cierto.<br>d. Totalmente cierto.<br>e. No he hecho esta actividad.
12013	ActivityLimitation	Me cuesta hacer tareas cotidianas, como limpiar, hacer la compra o cocinar.	a. Totalmente falsa.<br>b. Bastante falsa.<br>c. Bastante cierto.<br>d. Totalmente cierto.<br>e. No he hecho esta actividad.
12014	ActivityLimitation	Me cuesta hacerme cargo de niños.	a. Totalmente falsa.<br>b. Bastante falsa.<br>c. Bastante cierto.<br>d. Totalmente cierto.<br>e. No he hecho esta actividad.
12015	ActivityLimitation	Me cuesta leer algo que yo mismo he escrito.	a. Totalmente falsa.<br>b. Bastante falsa.<br>c. Bastante cierto.<br>d. Totalmente cierto.<br>e. No he hecho esta actividad.
12016	ActivityLimitation	Me cuesta leer las etiquetas de la ropa.	a. Totalmente falsa.<br>b. Bastante falsa.<br>c. Bastante cierto.<br>d. Totalmente cierto.<br>e. No he hecho esta actividad.
12017	ActivityLimitation	Me cuesta leer letra pequeña, como el prospecto de un medicamento.	a. Totalmente falsa.<br>b. Bastante falsa.<br>c. Bastante cierto.<br>d. Totalmente cierto.<br>e. No he hecho esta actividad.
12018	ActivityLimitation	Me cuesta leer letreros de la calle, como indicaciones o nombres de calles.	a. Totalmente falsa.<br>b. Bastante falsa.<br>c. Bastante cierto.<br>d. Totalmente cierto.<br>e. No he hecho esta actividad.

12019	ActivityLimitation	Me cuesta leer letreros luminosos en la calle.	a. Totalmente falsa.<br>b. Bastante falsa.<br>c. Bastante cierto.<br>d. Totalmente cierto.<br>e. No he hecho esta actividad.
12020	ActivityLimitation	Me cuesta leer un libro.	a. Totalmente falsa.<br>b. Bastante falsa.<br>c. Bastante cierto.<br>d. Totalmente cierto.<br>e. No he hecho esta actividad.
12021	ActivityLimitation	Me cuesta maquillarme.	a. Totalmente falsa.<br>b. Bastante falsa.<br>c. Bastante cierto.<br>d. Totalmente cierto.<br>e. No he hecho esta actividad.
12022	ActivityLimitation	Me cuesta nadar.	a. Totalmente falsa.<br>b. Bastante falsa.<br>c. Bastante cierto.<br>d. Totalmente cierto.<br>e. No he hecho esta actividad.
12023	ActivityLimitation	Me cuesta peinarme.	a. Totalmente falsa.<br>b. Bastante falsa.<br>c. Bastante cierto.<br>d. Totalmente cierto.<br>e. No he hecho esta actividad.
12024	ActivityLimitation	Me cuesta reconocer la cara de personas conocidas.	a. Totalmente falsa.<br>b. Bastante falsa.<br>c. Bastante cierto.<br>d. Totalmente cierto.<br>e. No he hecho esta actividad.
12025	ActivityLimitation	Me cuesta seguir una reunión de trabajo.	a. Totalmente falsa.<br>b. Bastante falsa.<br>c. Bastante cierto.<br>d. Totalmente cierto.<br>e. No he hecho esta actividad.
12026	ActivityLimitation	Me cuesta subir o bajar escaleras.	a. Totalmente falsa.<br>b. Bastante falsa.<br>c. Bastante cierto.<br>d. Totalmente cierto.<br>e. No he hecho esta actividad.

12027	ActivityLimitation	Me cuesta usar el móvil.	a. Totalmente falsa.<br>b. Bastante falsa.<br>c. Bastante cierto.<br>d. Totalmente cierto.<br>e. No he hecho esta actividad.
12028	ActivityLimitation	Me cuesta usar el teléfono fijo.	a. Totalmente falsa.<br>b. Bastante falsa.<br>c. Bastante cierto.<br>d. Totalmente cierto.<br>e. No he hecho esta actividad.
12029	ActivityLimitation	Me cuesta ver el ordenador.	a. Totalmente falsa.<br>b. Bastante falsa.<br>c. Bastante cierto.<br>d. Totalmente cierto.<br>e. No he hecho esta actividad.
12030	ActivityLimitation	Me cuesta ver la hora en un reloj de pared.	a. Totalmente falsa.<br>b. Bastante falsa.<br>c. Bastante cierto.<br>d. Totalmente cierto.<br>e. No he hecho esta actividad.
12031	ActivityLimitation	Me cuesta ver la hora en un reloj de pulsera.	a. Totalmente falsa.<br>b. Bastante falsa.<br>c. Bastante cierto.<br>d. Totalmente cierto.<br>e. No he hecho esta actividad.
12032	ActivityLimitation	Me cuesta ver la televisión.	a. Totalmente falsa.<br>b. Bastante falsa.<br>c. Bastante cierto.<br>d. Totalmente cierto.<br>e. No he hecho esta actividad.
12033	ActivityLimitation	Me cuesta ver una obra de teatro o una película en el cine.	a. Totalmente falsa.<br>b. Bastante falsa.<br>c. Bastante cierto.<br>d. Totalmente cierto.<br>e. No he hecho esta actividad.
12035	ActivityLimitation	Me cuesta viajar en transporte público.	a. Totalmente falsa.<br>b. Bastante falsa.<br>c. Bastante cierto.<br>d. Totalmente cierto.<br>e. No he hecho esta actividad.

12036	ActivityLimitation	Mientras hablo con alguien, me cuesta ver sus reacciones.	a. Totalmente falsa.<br>b. Bastante falsa.<br>c. Bastante cierto.<br>d. Totalmente cierto.<br>e. No he hecho esta actividad.
12037	ActivityLimitation	Me cuesta leer prospectos	a. Totalmente falsa.<br>b. Bastante falsa.<br>c. Bastante cierto.<br>d. Totalmente cierto.<br>e. No he hecho esta actividad.

id	trait	¿Cómo de cierta es la siguiente afirmación para usted?	
20001	Cognitive	Me cuesta tener mis cosas organizadas.	a. Totalmente falsa.<br>b. Bastante falsa.<br>c. Bastante cierto.<br>d. Totalmente cierto.
20002	Cognitive	Me cuesta organizar mi tiempo para hacer las actividades de mi día a día.	a. Totalmente falsa.<br>b. Bastante falsa.<br>c. Bastante cierto.<br>d. Totalmente cierto.
20003	Cognitive	Tengo despistes, como dejarme las llaves en casa.	a. Totalmente falsa.<br>b. Bastante falsa.<br>c. Bastante cierto.<br>d. Totalmente cierto.
20004	Cognitive	Me cuesta recordar dónde dejo las cosas, como las llaves.	a. Totalmente falsa.<br>b. Bastante falsa.<br>c. Bastante cierto.<br>d. Totalmente cierto.
20005	Cognitive	Me cuesta planificar cosas con días de antelación, como un viaje o una comida.	a. Totalmente falsa.<br>b. Bastante falsa.<br>c. Bastante cierto.<br>d. Totalmente cierto.
20006	Cognitive	Me cuesta recordar lo que leo.	a. Totalmente falsa.<br>b. Bastante falsa.<br>c. Bastante cierto.<br>d. Totalmente cierto.
20007	Cognitive	Me cuesta acudir a citas que no forman parte de mi rutina semanal. Por ejemplo, una cita con el dentista.	a. Totalmente falsa.<br>b. Bastante falsa.<br>c. Bastante cierto.<br>d. Totalmente cierto.
20008	Cognitive	Me cuesta recordar dónde guardo las cosas. Por ejemplo, algún documento importante.	a. Totalmente falsa.<br>b. Bastante falsa.<br>c. Bastante cierto.<br>d. Totalmente cierto.

20009	Cognitive	Me cuesta planificar los pasos de una tarea. Por ejemplo, mientras cocino.	a. Totalmente falsa.<br>b. Bastante falsa.<br>c. Bastante cierto.<br>d. Totalmente cierto.
20010	Cognitive	Afronto bien los problemas.	a. Totalmente falsa.<br>b. Bastante falsa.<br>c. Bastante cierto.<br>d. Totalmente cierto.
20011	Cognitive	Siento interés por hacer cosas nuevas.	a. Totalmente falsa.<br>b. Bastante falsa.<br>c. Bastante cierto.<br>d. Totalmente cierto.
20012	Cognitive	Me juzgo a mí mismo/a con dureza cuando cometo errores.	a. Totalmente falsa.<br>b. Bastante falsa.<br>c. Bastante cierto.<br>d. Totalmente cierto.
20013	Cognitive	Soy capaz de pensar con claridad.	a. Totalmente falsa.<br>b. Bastante falsa.<br>c. Bastante cierto.<br>d. Totalmente cierto.
20014	Cognitive	Soy capaz de tomar cualquier decisión.	a. Totalmente falsa.<br>b. Bastante falsa.<br>c. Bastante cierto.<br>d. Totalmente cierto.
20015	Cognitive	Me esfuerzo por ser una persona justa en mis decisiones.	a. Totalmente falsa.<br>b. Bastante falsa.<br>c. Bastante cierto.<br>d. Totalmente cierto.
20016	Cognitive	Me gusta planificar mis actividades de antemano.	a. Totalmente falsa.<br>b. Bastante falsa.<br>c. Bastante cierto.<br>d. Totalmente cierto.
20017	Cognitive	Disfruto siguiendo el protocolo social.	a. Totalmente falsa.<br>b. Bastante falsa.<br>c. Bastante cierto.<br>d. Totalmente cierto.
20018	Cognitive	Considero importante mantener una buena reputación.	a. Totalmente falsa.<br>b. Bastante falsa.<br>c. Bastante cierto.<br>d. Totalmente cierto.
20019	Cognitive	Siento la necesidad de cumplir con las expectativas de los demás.	a. Totalmente falsa.<br>b. Bastante falsa.<br>c. Bastante cierto.<br>d. Totalmente cierto.
20020	Cognitive	Me siento incómodo/a cuando veo a otras personas violando normas o reglas.	a. Totalmente falsa.<br>b. Bastante falsa.<br>c. Bastante cierto.<br>d. Totalmente cierto.

20021	Cognitive	Siento remordimientos si no cumplo con mis propias expectativas o estándares.	a. Totalmente falsa.<br>b. Bastante falsa.<br>c. Bastante cierto.<br>d. Totalmente cierto.
id	trait	¿Cómo de cierta es la siguiente afirmación para usted?	
33001	Convenience	Usando lentillas, tengo menos limitaciones en mi vida diaria.	a. Totalmente falsa.<br>b. Bastante falsa.<br>c. Bastante cierto.<br>d. Totalmente cierto.
33002	Convenience	Me resulta incómodo tener que alejar y acercar objetos para enfocar lo que quiero ver.	a. Totalmente falsa.<br>b. Bastante falsa.<br>c. Bastante cierto.<br>d. Totalmente cierto.
33003	Convenience	Me molesta llevar lentillas en días lluviosos.	a. Totalmente falsa.<br>b. Bastante falsa.<br>c. Bastante cierto.<br>d. Totalmente cierto.
33004	Convenience	Me cuesta adaptarme a lentillas nuevas.	a. Totalmente falsa.<br>b. Bastante falsa.<br>c. Bastante cierto.<br>d. Totalmente cierto.
33005	Convenience	Me gusta usar lentillas porque puedo llevar cualquier gafa de sol.	a. Totalmente falsa.<br>b. Bastante falsa.<br>c. Bastante cierto.<br>d. Totalmente cierto.
33006	Convenience	Pierdo interés en el uso de lentillas por el tiempo que invierto en su mantenimiento y limpieza.	a. Totalmente falsa.<br>b. Bastante falsa.<br>c. Bastante cierto.<br>d. Totalmente cierto.
33007	Convenience	Me quedo dormido con las lentillas puestas.	a. Totalmente falsa.<br>b. Bastante falsa.<br>c. Bastante cierto.<br>d. Totalmente cierto.
33008	Convenience	Me siento satisfecho con mi imagen usando lentillas.	a. Totalmente falsa.<br>b. Bastante falsa.<br>c. Bastante cierto.<br>d. Totalmente cierto.
33010	Convenience	Las lentillas se han convertido en algo imprescindible en mi vida diaria.	a. Totalmente falsa.<br>b. Bastante falsa.<br>c. Bastante cierto.<br>d. Totalmente cierto.
33011	Convenience	Me siento cómodo/a con mis lentillas.	a. Totalmente falsa.<br>b. Bastante falsa.<br>c. Bastante cierto.<br>d. Totalmente cierto.

33012	Convenience	Cuando voy a hacer alguna cosa, pienso siempre en si podré hacerla con mis lentillas.	a. Totalmente falsa.<br>b. Bastante falsa.<br>c. Bastante cierto.<br>d. Totalmente cierto.
33013	Convenience	Me es incómodo tener que cargar siempre con estuches y líquidos.	a. Totalmente falsa.<br>b. Bastante falsa.<br>c. Bastante cierto.<br>d. Totalmente cierto.
33014	Convenience	Siento que el uso de lentillas me limita.	a. Totalmente falsa.<br>b. Bastante falsa.<br>c. Bastante cierto.<br>d. Totalmente cierto.
34001	Convenience	Llevando las lentillas puestas, en ocasiones, me pongo unas gafas a la vez, para ver mejor.	a. Totalmente falsa.<br>b. Bastante falsa.<br>c. Bastante cierto.<br>d. Totalmente cierto.
31001	Convenience	Tengo gafas en todos sitios.	a. Totalmente falsa.<br>b. Bastante falsa.<br>c. Bastante cierto.<br>d. Totalmente cierto.
31002	Convenience	Me resulta incómodo tener que alejar y acercar objetos para enfocar lo que quiero ver.	a. Totalmente falsa.<br>b. Bastante falsa.<br>c. Bastante cierto.<br>d. Totalmente cierto.
31003	Convenience	Me molesta llevar gafas en días lluviosos.	a. Totalmente falsa.<br>b. Bastante falsa.<br>c. Bastante cierto.<br>d. Totalmente cierto.
31004	Convenience	Me cuesta adaptarme a gafas nuevas.	a. Totalmente falsa.<br>b. Bastante falsa.<br>c. Bastante cierto.<br>d. Totalmente cierto.
31005	Convenience	Me quedo dormido con las gafas puestas.	a. Totalmente falsa.<br>b. Bastante falsa.<br>c. Bastante cierto.<br>d. Totalmente cierto.
31006	Convenience	Olvido limpiar mis gafas.	a. Totalmente falsa.<br>b. Bastante falsa.<br>c. Bastante cierto.<br>d. Totalmente cierto.
31007	Convenience	Me siento satisfecho con mi imagen usando gafas.	a. Totalmente falsa.<br>b. Bastante falsa.<br>c. Bastante cierto.<br>d. Totalmente cierto.
31008	Convenience	Me resulta incómodo no ver bien al despertarme antes de ponerme las gafas.	a. Totalmente falsa.<br>b. Bastante falsa.<br>c. Bastante cierto.<br>d. Totalmente cierto.

31009	Convenience	Las gafas se han convertido en algo imprescindible en mi vida diaria.	a. Totalmente falsa.<br>b. Bastante falsa.<br>c. Bastante cierto.<br>d. Totalmente cierto.
31010	Convenience	Ser usuario de gafas me limita a la hora de elegir gafa de sol.	a. Totalmente falsa.<br>b. Bastante falsa.<br>c. Bastante cierto.<br>d. Totalmente cierto.
31011	Convenience	Me siento cómodo/a con mis gafas.	a. Totalmente falsa.<br>b. Bastante falsa.<br>c. Bastante cierto.<br>d. Totalmente cierto.
31012	Convenience	Cuando voy a hacer alguna cosa, pienso siempre en si podré hacerla con mis gafas.	a. Totalmente falsa.<br>b. Bastante falsa.<br>c. Bastante cierto.<br>d. Totalmente cierto.
31013	Convenience	Me es incómodo tener que ponerme y quitarme las gafas constantemente.	a. Totalmente falsa.<br>b. Bastante falsa.<br>c. Bastante cierto.<br>d. Totalmente cierto.
31014	Convenience	Siento que el uso de gafas me limita.	a. Totalmente falsa.<br>b. Bastante falsa.<br>c. Bastante cierto.<br>d. Totalmente cierto.
32001	Convenience	Me resulta incomodo tener que alejar y acercar objetos para enfocar lo que quiero ver.	a. Totalmente falsa.<br>b. Bastante falsa.<br>c. Bastante cierto.<br>d. Totalmente cierto.
32002	Convenience	Me resulta incomodo no ver bien al despertarme.	a. Totalmente falsa.<br>b. Bastante falsa.<br>c. Bastante cierto.<br>d. Totalmente cierto.
31015	Convenience	Llevar gafas mientras uso mascarilla me resulta molesto	a. Totalmente falsa.<br>b. Bastante falsa.<br>c. Bastante cierto.<br>d. Totalmente cierto.
31016	Convenience	Me preocupa la protección de mis ojos frente a la radiación ultravioleta	a. Totalmente falsa.<br>b. Bastante falsa.<br>c. Bastante cierto.<br>d. Totalmente cierto.
31017	Convenience	Me preocupa romper mis gafas y no tener otras cerca.	a. Totalmente falsa.<br>b. Bastante falsa.<br>c. Bastante cierto.<br>d. Totalmente cierto.
33015	Convenience	Usando lentillas, me preocupa no poder llevarlas todo el tiempo que necesito.	a. Totalmente falsa.<br>b. Bastante falsa.<br>c. Bastante cierto.<br>d. Totalmente cierto.

33016	Convenience	Me molesta tener que acudir a revisiones para ajustar mis lentillas	a. Totalmente falsa.<br>b. Bastante falsa.<br>c. Bastante cierto.<br>d. Totalmente cierto.
33017	Convenience	Me preocupa que se me caigan las lentillas y no tener conmigo otro método de corrección	a. Totalmente falsa.<br>b. Bastante falsa.<br>c. Bastante cierto.<br>d. Totalmente cierto.

id	trait		
43001	Economic Issues	¿Los gastos asociados al cuidado y mantenimiento de las lentillas (líquidos, estuches, etc.) son un factor importante a tener en cuenta al decidir a usarlas?	a.No.<br>b. Sí, poco.<br>c. Sí, bastante.<br>d. Sí, mucho.
43003	Economic Issues	¿Cómo de cierta es la siguiente afirmación para usted? Cuando elijo lentillas, priorizo la calidad por encima del precio.	a. Totalmente falsa.<br>b. Bastante falsa.<br>c. Bastante cierto.<br>d. Totalmente cierto.
43004	Economic Issues	¿En que medida considera que los beneficios de las lentillas progresivas justifican su coste?	a.No.<br>b. Sí, poco.<br>c. Sí, bastante.<br>d. Sí, mucho.
42001	Economic Issues	¿Considera el precio como un factor determinante al elegir que corrección (lentillas, gafas, etc.) para presbicia utilizar?	a.No.<br>b. Sí, poco.<br>c. Sí, bastante.<br>d. Sí, mucho.
42002	Economic Issues	¿Cómo de cierta es la siguiente afirmación para usted? Considero que el gasto que hago en productos para la salud visual (lentillas, gafas, etc.) es elevado.	a. Totalmente falsa.<br>b. Bastante falsa.<br>c. Bastante cierto.<br>d. Totalmente cierto.
43005	Economic Issues	Intento aprovechar al máximo mis lentillas, en ocasiones, alargando el tiempo de uso más de un mes.	a. Nunca<br>b. Raramente.<br>c. Frecuentemente.<br>d. Siempre.
41001	Economic Issues	¿Cómo de cierta es la siguiente afirmación para usted? Compro gafas con frecuencia porque disfruto de tener distintos pares.	a. Totalmente falsa.<br>b. Bastante falsa.<br>c. Bastante cierto.<br>d. Totalmente cierto.
43006	Economic Issues	¿Cómo de cierta es la siguiente afirmación para usted? Me preocupa tener que comprar lentillas nuevas en caso de rotura o pérdida.	a. Totalmente falsa.<br>b. Bastante falsa.<br>c. Bastante cierto.<br>d. Totalmente cierto.
41002	Economic Issues	¿Cómo de cierta es la siguiente afirmación para usted? Me preocupa tener que comprar gafas nuevas en caso de rotura o pérdida.	a. Totalmente falsa.<br>b. Bastante falsa.<br>c. Bastante cierto.<br>d. Totalmente cierto.

44001	Economic Issues	¿Cómo de cierta es la siguiente afirmación para usted? Considero aceptable hacer un gasto anual mayor en lentillas que en gafas	a. Totalmente falsa.<br>b. Bastante falsa.<br>c. Bastante cierto.<br>d. Totalmente cierto.
id	trait	¿Cómo de cierta es la siguiente afirmación para usted?	
50003	Emotional Well-Bei	Me siento animado/a a aprender cosas nuevas. Por ejemplo, aprender a ponerme las lentillas.	a. Totalmente falsa.<br>b. Bastante falsa.<br>c. Bastante cierto.<br>d. Totalmente cierto.
50010	Emotional Well-Bei	Me siento útil.	a. Totalmente falsa.<br>b. Bastante falsa.<br>c. Bastante cierto.<br>d. Totalmente cierto.
50012	Emotional Well-Bei	Me he sentido alegre.	a. Totalmente falsa.<br>b. Bastante falsa.<br>c. Bastante cierto.<br>d. Totalmente cierto.
50013	Emotional Well-Bei	Me siento relajado.	a. Totalmente falsa.<br>b. Bastante falsa.<br>c. Bastante cierto.<br>d. Totalmente cierto.
50014	Emotional Well-Bei	Me siento con confianza.	a. Totalmente falsa.<br>b. Bastante falsa.<br>c. Bastante cierto.<br>d. Totalmente cierto.
50015	Emotional Well-Bei	Me siento bien conmigo mismo/a.	a. Totalmente falsa.<br>b. Bastante falsa.<br>c. Bastante cierto.<br>d. Totalmente cierto.
50018	Emotional Well-Bei	Me siento cercano a los demás.	a. Totalmente falsa.<br>b. Bastante falsa.<br>c. Bastante cierto.<br>d. Totalmente cierto.
50019	Emotional Well-Bei	Me siento optimista respecto a mi futuro.	a. Totalmente falsa.<br>b. Bastante falsa.<br>c. Bastante cierto.<br>d. Totalmente cierto.
50020	Emotional Well-Bei	Me siento interesado por los demás.	a. Totalmente falsa.<br>b. Bastante falsa.<br>c. Bastante cierto.<br>d. Totalmente cierto.
54009	Emotional Well-Bei	Me desagrada la idea de depender de mis gafas y/o lentillas.	a. Totalmente falsa.<br>b. Bastante falsa.<br>c. Bastante cierto.<br>d. Totalmente cierto.

50001	Emotional Well-Bei	Me siento frustrado/a por no ver mejor.	a. Totalmente falsa.<br>b. Bastante falsa.<br>c. Bastante cierto.<br>d. Totalmente cierto.
50002	Emotional Well-Bei	Mi entorno me ayuda a buscar soluciones para mi vista cansada.	a. Totalmente falsa.<br>b. Bastante falsa.<br>c. Bastante cierto.<br>d. Totalmente cierto.
50004	Emotional Well-Bei	Debido a mi visión, me siento menos útil.	a. Totalmente falsa.<br>b. Bastante falsa.<br>c. Bastante cierto.<br>d. Totalmente cierto.
50005	Emotional Well-Bei	Me preocupa lo rápido que avanza mi vista cansada.	a. Totalmente falsa.<br>b. Bastante falsa.<br>c. Bastante cierto.<br>d. Totalmente cierto.
50006	Emotional Well-Bei	Me preocupa pensar que las lentillas podrían ser un riesgo para la salud de mis ojos.	a. Totalmente falsa.<br>b. Bastante falsa.<br>c. Bastante cierto.<br>d. Totalmente cierto.
50007	Emotional Well-Bei	Mi visión me hace sentir torpe.	a. Totalmente falsa.<br>b. Bastante falsa.<br>c. Bastante cierto.<br>d. Totalmente cierto.
51004	Emotional Well-Bei	Utilizar gafas para ver bien de cerca, me hace sentir mayor.	a. Totalmente falsa.<br>b. Bastante falsa.<br>c. Bastante cierto.<br>d. Totalmente cierto.
50009	Emotional Well-Bei	Me avergüenzo cuando no veo bien algo de cerca.	a. Totalmente falsa.<br>b. Bastante falsa.<br>c. Bastante cierto.<br>d. Totalmente cierto.
53001	Emotional Well-Bei	He sentido ilusión durante el proceso de adaptación de mis lentillas.	a. Totalmente falsa.<br>b. Bastante falsa.<br>c. Bastante cierto.<br>d. Totalmente cierto.
53002	Emotional Well-Bei	Me siento decepcionado/a porque con las lentillas no veo tan bien como me gustaría.	a. Totalmente falsa.<br>b. Bastante falsa.<br>c. Bastante cierto.<br>d. Totalmente cierto.
53003	Emotional Well-Bei	Usando lentillas, me siento más seguro de mí mismo.	a. Totalmente falsa.<br>b. Bastante falsa.<br>c. Bastante cierto.<br>d. Totalmente cierto.
53004	Emotional Well-Bei	Soy más feliz usando lentillas.	a. Totalmente falsa.<br>b. Bastante falsa.<br>c. Bastante cierto.<br>d. Totalmente cierto.

53005	Emotional Well-Bei	Usar lentillas me hace sentir más joven.	a. Totalmente falsa.<br>b. Bastante falsa.<br>c. Bastante cierto.<br>d. Totalmente cierto.
53006	Emotional Well-Bei	Usar lentillas me da una mayor sensación de libertad.	a. Totalmente falsa.<br>b. Bastante falsa.<br>c. Bastante cierto.<br>d. Totalmente cierto.
53007	Emotional Well-Bei	Me hace ilusión poder ver bien todas las distancias con lentillas.	a. Totalmente falsa.<br>b. Bastante falsa.<br>c. Bastante cierto.<br>d. Totalmente cierto.
53008	Emotional Well-Bei	Me preocupa no poder llevar las lentillas tantas horas como lo necesito.	a. Totalmente falsa.<br>b. Bastante falsa.<br>c. Bastante cierto.<br>d. Totalmente cierto.
53009	Emotional Well-Bei	Me produce inseguridad tener que depender de mis lentillas.	a. Totalmente falsa.<br>b. Bastante falsa.<br>c. Bastante cierto.<br>d. Totalmente cierto.
53010	Emotional Well-Bei	Me produce ansiedad la posibilidad de romper mis lentillas.	a. Totalmente falsa.<br>b. Bastante falsa.<br>c. Bastante cierto.<br>d. Totalmente cierto.
53011	Emotional Well-Bei	Me produce ansiedad la posibilidad de perder mis lentillas.	a. Totalmente falsa.<br>b. Bastante falsa.<br>c. Bastante cierto.<br>d. Totalmente cierto.
51001	Emotional Well-Bei	Me produce ansiedad perder mis gafas.	a. Totalmente falsa.<br>b. Bastante falsa.<br>c. Bastante cierto.<br>d. Totalmente cierto.
51005	Emotional Well-Bei	Me produce ansiedad la posibilidad de romper mis gafas.	a. Totalmente falsa.<br>b. Bastante falsa.<br>c. Bastante cierto.<br>d. Totalmente cierto.
51002	Emotional Well-Bei	Probar unas nuevas gafas me ilusiona.	a. Totalmente falsa.<br>b. Bastante falsa.<br>c. Bastante cierto.<br>d. Totalmente cierto.
51003	Emotional Well-Bei	Estoy satisfecho/a con las gafas que uso en la actualidad.	a. Totalmente falsa.<br>b. Bastante falsa.<br>c. Bastante cierto.<br>d. Totalmente cierto.
id	trait		

60001	GeneralSymptoms	¿Con qué frecuencia le da sueño durante el día?	a. Nunca<br>b. Raramente.<br>c. Frecuentemente.<br>d. Siempre.
60005	GeneralSymptoms	¿Con qué frecuencia le dan mareos?	a. Nunca<br>b. Raramente.<br>c. Frecuentemente.<br>d. Siempre.
60008	GeneralSymptoms	¿Con qué frecuencia le cuesta concentrarse?	a. Nunca<br>b. Raramente.<br>c. Frecuentemente.<br>d. Siempre.
60011	GeneralSymptoms	¿Con qué frecuencia le dan dolores de cabeza?	a. Nunca<br>b. Raramente.<br>c. Frecuentemente.<br>d. Siempre.
60014	GeneralSymptoms	¿Con qué frecuencia le dan dolores de cabeza tras estar fijando la vista en cerca?	a. Nunca<br>b. Raramente.<br>c. Frecuentemente.<br>d. Siempre.
60002	GeneralSymptoms	¿Le da sueño durante el día?	a.No.<br>b. Sí, poco.<br>c. Sí, bastante.<br>d. Sí, mucho.
60004	GeneralSymptoms	¿Le dan mareos?	a.No.<br>b. Sí, poco.<br>c. Sí, bastante.<br>d. Sí, mucho.
60007	GeneralSymptoms	¿Le cuesta concentrarse?	a.No.<br>b. Sí, poco.<br>c. Sí, bastante.<br>d. Sí, mucho.
60010	GeneralSymptoms	¿Le dan dolores de cabeza?	a.No.<br>b. Sí, poco.<br>c. Sí, bastante.<br>d. Sí, mucho.
60013	GeneralSymptoms	¿Le dan dolores de cabeza tras estar fijando la vista en cerca?	a.No.<br>b. Sí, poco.<br>c. Sí, bastante.<br>d. Sí, mucho.
		¿Cómo de cierta es la siguiente afirmación para usted?	
60016	GeneralSymptoms	Diría que su salud, en general, es:	a. Totalmente falsa.<br>b. Bastante falsa.<br>c. Bastante cierto.<br>d. Totalmente cierto.

60003	GeneralSymptoms	Me da sueño durante el día.	a. Totalmente falsa.<br>b. Bastante falsa.<br>c. Bastante cierto.<br>d. Totalmente cierto.
60006	GeneralSymptoms	Me dan mareos.	a. Totalmente falsa.<br>b. Bastante falsa.<br>c. Bastante cierto.<br>d. Totalmente cierto.
60009	GeneralSymptoms	Pierdo la concentración a lo largo del día.	a. Totalmente falsa.<br>b. Bastante falsa.<br>c. Bastante cierto.<br>d. Totalmente cierto.
60012	GeneralSymptoms	Me dan dolores de cabeza.	a. Totalmente falsa.<br>b. Bastante falsa.<br>c. Bastante cierto.<br>d. Totalmente cierto.
60015	GeneralSymptoms	Me dan dolores de cabeza tras haber estado fijando la vista en cerca.	a. Totalmente falsa.<br>b. Bastante falsa.<br>c. Bastante cierto.<br>d. Totalmente cierto.
id	trait	Usando gafas:	
71001	OcularSymptoms	¿Con qué frecuencia le lloran los ojos?	a. Nunca<br>b. Raramente.<br>c. Frecuentemente.<br>d. Siempre.
71004	OcularSymptoms	¿Con qué frecuencia tiene los ojos rojos?	a. Nunca<br>b. Raramente.<br>c. Frecuentemente.<br>d. Siempre.
71007	OcularSymptoms	¿Con qué frecuencia siente ardor en los ojos?	a. Nunca<br>b. Raramente.<br>c. Frecuentemente.<br>d. Siempre.
71010	OcularSymptoms	¿Con qué frecuencia le duelen los ojos?	a. Nunca<br>b. Raramente.<br>c. Frecuentemente.<br>d. Siempre.
71013	OcularSymptoms	¿Con qué frecuencia nota los ojos cansados?	a. Nunca<br>b. Raramente.<br>c. Frecuentemente.<br>d. Siempre.
71016	OcularSymptoms	¿Con qué frecuencia nota los ojos secos?	a. Nunca<br>b. Raramente.<br>c. Frecuentemente.<br>d. Siempre.

71019	OcularSymptoms	¿Con qué frecuencia le pican los ojos?	a. Nunca<br>b. Raramente.<br>c. Frecuentemente.<br>d. Siempre.
71022	OcularSymptoms	¿Con qué frecuencia despierta con legañas?	a. Nunca<br>b. Raramente.<br>c. Frecuentemente.<br>d. Siempre.
71025	OcularSymptoms	¿Con qué frecuencia nota los ojos secos cuando trabaja en el ordenador?	a. Nunca<br>b. Raramente.<br>c. Frecuentemente.<br>d. Siempre.
71028	OcularSymptoms	¿Con qué frecuencia siente como si tuviese arenilla en los ojos?	a. Nunca<br>b. Raramente.<br>c. Frecuentemente.<br>d. Siempre.
71031	OcularSymptoms	¿Con qué frecuencia cierra los ojos para aliviar la sensación de sequedad?	a. Nunca<br>b. Raramente.<br>c. Frecuentemente.<br>d. Siempre.
71034	OcularSymptoms	¿Con qué frecuencia siente molestias, en sus ojos, en ambientes secos?	a. Nunca<br>b. Raramente.<br>c. Frecuentemente.<br>d. Siempre.
71037	OcularSymptoms	¿Con qué frecuencia usa lágrima artificial?	a. Nunca<br>b. Raramente.<br>c. Frecuentemente.<br>d. Siempre.
71002	OcularSymptoms	¿Le lloran los ojos?	a.No.<br>b. Sí, poco.<br>c. Sí, bastante.<br>d. Sí, mucho.
71005	OcularSymptoms	¿Tiene los ojos rojos?	a.No.<br>b. Sí, poco.<br>c. Sí, bastante.<br>d. Sí, mucho.
71008	OcularSymptoms	¿Le arden los ojos?	a.No.<br>b. Sí, poco.<br>c. Sí, bastante.<br>d. Sí, mucho.
71011	OcularSymptoms	¿Le duelen los ojos?	a.No.<br>b. Sí, poco.<br>c. Sí, bastante.<br>d. Sí, mucho.
71014	OcularSymptoms	¿Nota los ojos cansados?	a.No.<br>b. Sí, poco.<br>c. Sí, bastante.<br>d. Sí, mucho.

71017	OcularSymptoms	¿Nota los ojos secos?	a.No.<br>b. Sí, poco.<br>c. Sí, bastante.<br>d. Sí, mucho.
71020	OcularSymptoms	¿Le pican los ojos?	a.No.<br>b. Sí, poco.<br>c. Sí, bastante.<br>d. Sí, mucho.
71023	OcularSymptoms	¿Despierta con legañas?	a.No.<br>b. Sí, poco.<br>c. Sí, bastante.<br>d. Sí, mucho.
71026	OcularSymptoms	¿Nota los ojos secos cuando trabaja en el ordenador?	a.No.<br>b. Sí, poco.<br>c. Sí, bastante.<br>d. Sí, mucho.
71029	OcularSymptoms	¿Siente como si tuviese arenilla en los ojos?	a.No.<br>b. Sí, poco.<br>c. Sí, bastante.<br>d. Sí, mucho.
71032	OcularSymptoms	¿Cierra los ojos para aliviar la sensación de sequedad?	a.No.<br>b. Sí, poco.<br>c. Sí, bastante.<br>d. Sí, mucho.
71035	OcularSymptoms	¿Le molestan sus ojos en ambientes secos?	a.No.<br>b. Sí, poco.<br>c. Sí, bastante.<br>d. Sí, mucho.
71038	OcularSymptoms	¿Usa lágrima artificial?	a.No.<br>b. Sí, poco.<br>c. Sí, bastante.<br>d. Sí, mucho.
		¿Cómo de cierta es la siguiente afirmación para usted? Usando gafas...	
71003	OcularSymptoms	Me lloran los ojos.	a. Totalmente falsa.<br>b. Bastante falsa.<br>c. Bastante cierto.<br>d. Totalmente cierto.
71006	OcularSymptoms	Tengo los ojos rojos.	a. Totalmente falsa.<br>b. Bastante falsa.<br>c. Bastante cierto.<br>d. Totalmente cierto.
71009	OcularSymptoms	Siento ardor los ojos.	a. Totalmente falsa.<br>b. Bastante falsa.<br>c. Bastante cierto.<br>d. Totalmente cierto.

71012	OcularSymptoms	Me duelen los ojos.	a. Totalmente falsa.<br>b. Bastante falsa.<br>c. Bastante cierto.<br>d. Totalmente cierto.
71015	OcularSymptoms	Noto los ojos cansados.	a. Totalmente falsa.<br>b. Bastante falsa.<br>c. Bastante cierto.<br>d. Totalmente cierto.
71018	OcularSymptoms	Noto los ojos secos.	a. Totalmente falsa.<br>b. Bastante falsa.<br>c. Bastante cierto.<br>d. Totalmente cierto.
71021	OcularSymptoms	Me pican los ojos.	a. Totalmente falsa.<br>b. Bastante falsa.<br>c. Bastante cierto.<br>d. Totalmente cierto.
71024	OcularSymptoms	Despierto con legañas.	a. Totalmente falsa.<br>b. Bastante falsa.<br>c. Bastante cierto.<br>d. Totalmente cierto.
71027	OcularSymptoms	Noto los ojos secos cuando trabajo en un ordenador.	a. Totalmente falsa.<br>b. Bastante falsa.<br>c. Bastante cierto.<br>d. Totalmente cierto.
71030	OcularSymptoms	Siento que tengo arenilla en los ojos.	a. Totalmente falsa.<br>b. Bastante falsa.<br>c. Bastante cierto.<br>d. Totalmente cierto.
71033	OcularSymptoms	Tengo que cerrar los ojos para aliviar la sensación de sequedad.	a. Totalmente falsa.<br>b. Bastante falsa.<br>c. Bastante cierto.<br>d. Totalmente cierto.
71036	OcularSymptoms	Siento molestias en los ojos en ambientes secos.	a. Totalmente falsa.<br>b. Bastante falsa.<br>c. Bastante cierto.<br>d. Totalmente cierto.
71039	OcularSymptoms	Uso lágrima artificial.	a. Totalmente falsa.<br>b. Bastante falsa.<br>c. Bastante cierto.<br>d. Totalmente cierto.

id	trait	Usando lentillas:	
73001	OcularSymptoms	¿Con qué frecuencia le lloran los ojos?	a. Nunca<br>b. Raramente.<br>c. Frecuentemente.<br>d. Siempre.

73004	OcularSymptoms	¿Con qué frecuencia tiene los ojos rojos?	a. Nunca<br>b. Raramente.<br>c. Frecuentemente.<br>d. Siempre.
73007	OcularSymptoms	¿Con qué frecuencia siente ardor en los ojos?	a. Nunca<br>b. Raramente.<br>c. Frecuentemente.<br>d. Siempre.
73010	OcularSymptoms	¿Con qué frecuencia le duelen los ojos?	a. Nunca<br>b. Raramente.<br>c. Frecuentemente.<br>d. Siempre.
73013	OcularSymptoms	¿Con qué frecuencia nota los ojos cansados?	a. Nunca<br>b. Raramente.<br>c. Frecuentemente.<br>d. Siempre.
73016	OcularSymptoms	¿Con qué frecuencia nota los ojos secos?	a. Nunca<br>b. Raramente.<br>c. Frecuentemente.<br>d. Siempre.
73019	OcularSymptoms	¿Con qué frecuencia le pican los ojos?	a. Nunca<br>b. Raramente.<br>c. Frecuentemente.<br>d. Siempre.
73022	OcularSymptoms	¿Con qué frecuencia despierta con legañas?	a. Nunca<br>b. Raramente.<br>c. Frecuentemente.<br>d. Siempre.
73025	OcularSymptoms	¿Con qué frecuencia nota los ojos secos cuando trabaja en el ordenador?	a. Nunca<br>b. Raramente.<br>c. Frecuentemente.<br>d. Siempre.
73028	OcularSymptoms	¿Con qué frecuencia siente como si tuviese arenilla en los ojos?	a. Nunca<br>b. Raramente.<br>c. Frecuentemente.<br>d. Siempre.
73031	OcularSymptoms	¿Con qué frecuencia cierra los ojos para aliviar la sensación de sequedad?	a. Nunca<br>b. Raramente.<br>c. Frecuentemente.<br>d. Siempre.
73034	OcularSymptoms	¿Con qué frecuencia siente molestias, en sus ojos, en ambientes secos?	a. Nunca<br>b. Raramente.<br>c. Frecuentemente.<br>d. Siempre.
73037	OcularSymptoms	¿Con qué frecuencia usa lágrima artificial?	a. Nunca<br>b. Raramente.<br>c. Frecuentemente.<br>d. Siempre.

73043	OcularSymptoms	¿Con qué frecuencia nota que las lentillas se mueven?	a. Nunca<br>b. Raramente.<br>c. Frecuentemente.<br>d. Siempre.
73046	OcularSymptoms	¿Con qué frecuencia se le quedan las lentillas pegadas a los ojos?	a. Nunca<br>b. Raramente.<br>c. Frecuentemente.<br>d. Siempre.
73049	OcularSymptoms	¿Con qué frecuencia nota molestias en los párpados?	a. Nunca<br>b. Raramente.<br>c. Frecuentemente.<br>d. Siempre.
73052	OcularSymptoms	¿Con qué frecuencia le cuesta ponerse las lentillas?	a. Nunca<br>b. Raramente.<br>c. Frecuentemente.<br>d. Siempre.
73053	OcularSymptoms	¿Con qué frecuencia le cuesta quitarse las lentillas?	a. Nunca<br>b. Raramente.<br>c. Frecuentemente.<br>d. Siempre.
73057	OcularSymptoms	¿Con qué frecuencia nota las lentillas secas en el ojo?	a. Nunca<br>b. Raramente.<br>c. Frecuentemente.<br>d. Siempre.
73002	OcularSymptoms	¿Le lloran los ojos?	a.No.<br>b. Sí, poco.<br>c. Sí, bastante.<br>d. Sí, mucho.
73005	OcularSymptoms	¿Tiene los ojos rojos?	a.No.<br>b. Sí, poco.<br>c. Sí, bastante.<br>d. Sí, mucho.
73008	OcularSymptoms	¿Le arden los ojos?	a.No.<br>b. Sí, poco.<br>c. Sí, bastante.<br>d. Sí, mucho.
73011	OcularSymptoms	¿Le duelen los ojos?	a.No.<br>b. Sí, poco.<br>c. Sí, bastante.<br>d. Sí, mucho.
73014	OcularSymptoms	¿Nota los ojos cansados?	a.No.<br>b. Sí, poco.<br>c. Sí, bastante.<br>d. Sí, mucho.
73017	OcularSymptoms	¿Nota los ojos secos?	a.No.<br>b. Sí, poco.<br>c. Sí, bastante.<br>d. Sí, mucho.

73020	OcularSymptoms	¿Le pican los ojos?	a.No.<br>b. Sí, poco.<br>c. Sí, bastante.<br>d. Sí, mucho.
73023	OcularSymptoms	¿Despierta con legañas?	a.No.<br>b. Sí, poco.<br>c. Sí, bastante.<br>d. Sí, mucho.
73026	OcularSymptoms	¿Nota los ojos secos cuando trabaja en el ordenador?	a.No.<br>b. Sí, poco.<br>c. Sí, bastante.<br>d. Sí, mucho.
73029	OcularSymptoms	¿Siente como si tuviese arenilla en los ojos?	a.No.<br>b. Sí, poco.<br>c. Sí, bastante.<br>d. Sí, mucho.
73032	OcularSymptoms	¿Cierra los ojos para aliviar la sensación de sequedad?	a.No.<br>b. Sí, poco.<br>c. Sí, bastante.<br>d. Sí, mucho.
73035	OcularSymptoms	¿Le molestan sus ojos en ambientes secos?	a.No.<br>b. Sí, poco.<br>c. Sí, bastante.<br>d. Sí, mucho.
73038	OcularSymptoms	¿Usa lágrima artificial?	a.No.<br>b. Sí, poco.<br>c. Sí, bastante.<br>d. Sí, mucho.
73041	OcularSymptoms	¿Nota que las lentillas se le mueven?	a.No.<br>b. Sí, poco.<br>c. Sí, bastante.<br>d. Sí, mucho.
73044	OcularSymptoms	¿Se le quedan las lentillas pegadas a los ojos?	a.No.<br>b. Sí, poco.<br>c. Sí, bastante.<br>d. Sí, mucho.
73047	OcularSymptoms	¿Nota molestias en los párpados?	a.No.<br>b. Sí, poco.<br>c. Sí, bastante.<br>d. Sí, mucho.
73050	OcularSymptoms	¿Le cuesta ponerse las lentillas?	a.No.<br>b. Sí, poco.<br>c. Sí, bastante.<br>d. Sí, mucho.
73055	OcularSymptoms	¿Le cuesta quitarse las lentillas?	a.No.<br>b. Sí, poco.<br>c. Sí, bastante.<br>d. Sí, mucho.

73058	OcularSymptoms	¿Nota las lentillas secas en el ojo?	a.No.<br>b. Sí, poco.<br>c. Sí, bastante.<br>d. Sí, mucho.
		¿Cómo de cierta es la siguiente afirmación para usted? Usando lentillas...	
73003	OcularSymptoms	Me lloran los ojos.	a. Totalmente falsa.<br>b. Bastante falsa.<br>c. Bastante cierto.<br>d. Totalmente cierto.
73006	OcularSymptoms	Tengo los ojos rojos.	a. Totalmente falsa.<br>b. Bastante falsa.<br>c. Bastante cierto.<br>d. Totalmente cierto.
73009	OcularSymptoms	Siento ardor en los ojos.	a. Totalmente falsa.<br>b. Bastante falsa.<br>c. Bastante cierto.<br>d. Totalmente cierto.
73012	OcularSymptoms	Me duelen los ojos.	a. Totalmente falsa.<br>b. Bastante falsa.<br>c. Bastante cierto.<br>d. Totalmente cierto.
73015	OcularSymptoms	Noto los ojos cansados.	a. Totalmente falsa.<br>b. Bastante falsa.<br>c. Bastante cierto.<br>d. Totalmente cierto.
73018	OcularSymptoms	Noto los ojos secos.	a. Totalmente falsa.<br>b. Bastante falsa.<br>c. Bastante cierto.<br>d. Totalmente cierto.
73021	OcularSymptoms	Me pican los ojos.	a. Totalmente falsa.<br>b. Bastante falsa.<br>c. Bastante cierto.<br>d. Totalmente cierto.
73024	OcularSymptoms	Despierto con legañas.	a. Totalmente falsa.<br>b. Bastante falsa.<br>c. Bastante cierto.<br>d. Totalmente cierto.
73027	OcularSymptoms	Noto los ojos secos cuando trabajo en un ordenador.	a. Totalmente falsa.<br>b. Bastante falsa.<br>c. Bastante cierto.<br>d. Totalmente cierto.
73030	OcularSymptoms	Siento que tengo arenilla en los ojos.	a. Totalmente falsa.<br>b. Bastante falsa.<br>c. Bastante cierto.<br>d. Totalmente cierto.

73033	OcularSymptoms	Tengo que cerrar los ojos para aliviar la sensación de sequedad.	a. Totalmente falsa.<br>b. Bastante falsa.<br>c. Bastante cierto.<br>d. Totalmente cierto.
73036	OcularSymptoms	Siento molestias en los ojos en ambientes secos.	a. Totalmente falsa.<br>b. Bastante falsa.<br>c. Bastante cierto.<br>d. Totalmente cierto.
73039	OcularSymptoms	Uso lágrima artificial.	a. Totalmente falsa.<br>b. Bastante falsa.<br>c. Bastante cierto.<br>d. Totalmente cierto.
73042	OcularSymptoms	Noto que las lentillas se mueven.	a. Totalmente falsa.<br>b. Bastante falsa.<br>c. Bastante cierto.<br>d. Totalmente cierto.
73048	OcularSymptoms	Siento que las lentillas se pegan a mis ojos.	a. Totalmente falsa.<br>b. Bastante falsa.<br>c. Bastante cierto.<br>d. Totalmente cierto.
73051	OcularSymptoms	Siento molestias en los párpados.	a. Totalmente falsa.<br>b. Bastante falsa.<br>c. Bastante cierto.<br>d. Totalmente cierto.
73054	OcularSymptoms	Me cuesta ponerme las lentillas.	a. Totalmente falsa.<br>b. Bastante falsa.<br>c. Bastante cierto.<br>d. Totalmente cierto.
73056	OcularSymptoms	Me cuesta quitarme las lentillas.	a. Totalmente falsa.<br>b. Bastante falsa.<br>c. Bastante cierto.<br>d. Totalmente cierto.
73059	OcularSymptoms	Noto las lentillas secas en el ojo.	a. Totalmente falsa.<br>b. Bastante falsa.<br>c. Bastante cierto.<br>d. Totalmente cierto.
id	trait	Sin usar gafas o lentillas:	
72001	OcularSymptoms	¿Con qué frecuencia le lloran los ojos?	a. Nunca<br>b. Raramente.<br>c. Frecuentemente.<br>d. Siempre.
72004	OcularSymptoms	¿Con qué frecuencia tiene los ojos rojos?	a. Nunca<br>b. Raramente.<br>c. Frecuentemente.<br>d. Siempre.

72007	OcularSymptoms	¿Con qué frecuencia siente ardor en los ojos?	a. Nunca<br>b. Raramente.<br>c. Frecuentemente.<br>d. Siempre.
72010	OcularSymptoms	¿Con qué frecuencia le duelen los ojos?	a. Nunca<br>b. Raramente.<br>c. Frecuentemente.<br>d. Siempre.
72013	OcularSymptoms	¿Con qué frecuencia nota los ojos cansados?	a. Nunca<br>b. Raramente.<br>c. Frecuentemente.<br>d. Siempre.
72016	OcularSymptoms	¿Con qué frecuencia nota los ojos secos?	a. Nunca<br>b. Raramente.<br>c. Frecuentemente.<br>d. Siempre.
72019	OcularSymptoms	¿Con qué frecuencia le pican los ojos?	a. Nunca<br>b. Raramente.<br>c. Frecuentemente.<br>d. Siempre.
72022	OcularSymptoms	¿Con qué frecuencia despierta con legañas?	a. Nunca<br>b. Raramente.<br>c. Frecuentemente.<br>d. Siempre.
72025	OcularSymptoms	¿Con qué frecuencia nota los ojos secos cuando trabaja en el ordenador?	a. Nunca<br>b. Raramente.<br>c. Frecuentemente.<br>d. Siempre.
72028	OcularSymptoms	¿Con qué frecuencia siente como si tuviese arenilla en los ojos?	a. Nunca<br>b. Raramente.<br>c. Frecuentemente.<br>d. Siempre.
72031	OcularSymptoms	¿Con qué frecuencia cierra los ojos para aliviar la sensación de sequedad?	a. Nunca<br>b. Raramente.<br>c. Frecuentemente.<br>d. Siempre.
72034	OcularSymptoms	¿Con qué frecuencia siente molestias, en sus ojos, en ambientes secos?	a. Nunca<br>b. Raramente.<br>c. Frecuentemente.<br>d. Siempre.
72037	OcularSymptoms	¿Con qué frecuencia usa lágrima artificial?	a. Nunca<br>b. Raramente.<br>c. Frecuentemente.<br>d. Siempre.
72002	OcularSymptoms	¿Le lloran los ojos?	a.No.<br>b. Sí, poco.<br>c. Sí, bastante.<br>d. Sí, mucho.

72005	OcularSymptoms	¿Tiene los ojos rojos?	a.No.<br>b. Sí, poco.<br>c. Sí, bastante.<br>d. Sí, mucho.
72008	OcularSymptoms	¿Le arden los ojos?	a.No.<br>b. Sí, poco.<br>c. Sí, bastante.<br>d. Sí, mucho.
72011	OcularSymptoms	¿Le duelen los ojos?	a.No.<br>b. Sí, poco.<br>c. Sí, bastante.<br>d. Sí, mucho.
72014	OcularSymptoms	¿Nota los ojos cansados?	a.No.<br>b. Sí, poco.<br>c. Sí, bastante.<br>d. Sí, mucho.
72017	OcularSymptoms	¿Nota los ojos secos?	a.No.<br>b. Sí, poco.<br>c. Sí, bastante.<br>d. Sí, mucho.
72020	OcularSymptoms	¿Le pican los ojos?	a.No.<br>b. Sí, poco.<br>c. Sí, bastante.<br>d. Sí, mucho.
72023	OcularSymptoms	¿Despierta con legañas?	a.No.<br>b. Sí, poco.<br>c. Sí, bastante.<br>d. Sí, mucho.
72026	OcularSymptoms	¿Nota los ojos secos cuando trabaja en el ordenador?	a.No.<br>b. Sí, poco.<br>c. Sí, bastante.<br>d. Sí, mucho.
72029	OcularSymptoms	¿Siente como si tuviese arenilla en los ojos?	a.No.<br>b. Sí, poco.<br>c. Sí, bastante.<br>d. Sí, mucho.
72032	OcularSymptoms	¿Cierra los ojos para aliviar la sensación de sequedad?	a.No.<br>b. Sí, poco.<br>c. Sí, bastante.<br>d. Sí, mucho.
72035	OcularSymptoms	¿Le molestan sus ojos en ambientes secos?	a.No.<br>b. Sí, poco.<br>c. Sí, bastante.<br>d. Sí, mucho.
72038	OcularSymptoms	¿Usa lágrima artificial?	a.No.<br>b. Sí, poco.<br>c. Sí, bastante.<br>d. Sí, mucho.

		¿Cómo de cierta es la siguiente afirmación para usted? Sin usar gafas ni lentillas…	
72003	OcularSymptoms	Me lloran los ojos.	a. Totalmente falsa.<br>b. Bastante falsa.<br>c. Bastante cierto.<br>d. Totalmente cierto.
72006	OcularSymptoms	Tengo los ojos rojos.	a. Totalmente falsa.<br>b. Bastante falsa.<br>c. Bastante cierto.<br>d. Totalmente cierto.
72009	OcularSymptoms	Siento ardor en los ojos.	a. Totalmente falsa.<br>b. Bastante falsa.<br>c. Bastante cierto.<br>d. Totalmente cierto.
72012	OcularSymptoms	Me duelen los ojos.	a. Totalmente falsa.<br>b. Bastante falsa.<br>c. Bastante cierto.<br>d. Totalmente cierto.
72015	OcularSymptoms	Noto los ojos cansados.	a. Totalmente falsa.<br>b. Bastante falsa.<br>c. Bastante cierto.<br>d. Totalmente cierto.
72018	OcularSymptoms	Noto los ojos secos.	a. Totalmente falsa.<br>b. Bastante falsa.<br>c. Bastante cierto.<br>d. Totalmente cierto.
72021	OcularSymptoms	Me pican los ojos.	a. Totalmente falsa.<br>b. Bastante falsa.<br>c. Bastante cierto.<br>d. Totalmente cierto.
72024	OcularSymptoms	Despierto con legañas.	a. Totalmente falsa.<br>b. Bastante falsa.<br>c. Bastante cierto.<br>d. Totalmente cierto.
72027	OcularSymptoms	Noto los ojos secos cuando trabajo en un ordenador.	a. Totalmente falsa.<br>b. Bastante falsa.<br>c. Bastante cierto.<br>d. Totalmente cierto.
72030	OcularSymptoms	Siento que tengo arenilla en los ojos.	a. Totalmente falsa.<br>b. Bastante falsa.<br>c. Bastante cierto.<br>d. Totalmente cierto.
72033	OcularSymptoms	Tengo que cerrar los ojos para aliviar la sensación de sequedad.	a. Totalmente falsa.<br>b. Bastante falsa.<br>c. Bastante cierto.<br>d. Totalmente cierto.

72036	OcularSymptoms	Siento molestias en los ojos en ambientes secos.	a. Totalmente falsa.<br>b. Bastante falsa.<br>c. Bastante cierto.<br>d. Totalmente cierto.
72039	OcularSymptoms	Uso lágrima artificial.	a. Totalmente falsa.<br>b. Bastante falsa.<br>c. Bastante cierto.<br>d. Totalmente cierto.
id	trait	Usando gafas:	
81001	VisualSymptoms	¿Con qué frecuencia ve borroso al mirar de lejos?	a. Nunca<br>b. Raramente.<br>c. Frecuentemente.<br>d. Siempre.
81004	VisualSymptoms	¿Con qué frecuencia ve borroso al mirar de cerca?	a. Nunca<br>b. Raramente.<br>c. Frecuentemente.<br>d. Siempre.
81007	VisualSymptoms	¿Con qué frecuencia ve borrosa la pantalla del ordenador?	a. Nunca<br>b. Raramente.<br>c. Frecuentemente.<br>d. Siempre.
81013	VisualSymptoms	¿Con qué frecuencia necesita entornar los ojos para ver bien?	a. Nunca<br>b. Raramente.<br>c. Frecuentemente.<br>d. Siempre.
81025	VisualSymptoms	¿Con qué frecuencia le molestan las luces?	a. Nunca<br>b. Raramente.<br>c. Frecuentemente.<br>d. Siempre.
81028	VisualSymptoms	¿Con qué frecuencia ve peor en ambientes poco iluminados?	a. Nunca<br>b. Raramente.<br>c. Frecuentemente.<br>d. Siempre.
81031	VisualSymptoms	¿Con qué frecuencia le molestan los ojos cuando hay mucha luz?	a. Nunca<br>b. Raramente.<br>c. Frecuentemente.<br>d. Siempre.
81034	VisualSymptoms	¿Con qué frecuencia ve peor en ambientes muy iluminados?	a. Nunca<br>b. Raramente.<br>c. Frecuentemente.<br>d. Siempre.
81037	VisualSymptoms	¿Con qué frecuencia le cuesta ver bien, cuando pasa de un ambiente muy iluminado a otro poco iluminado?	a. Nunca<br>b. Raramente.<br>c. Frecuentemente.<br>d. Siempre.

81040	VisualSymptoms	¿Con qué frecuencia ve mal los letreros luminosos de la calle? Por ejemplo, el letrero de una farmacia.	a. Nunca<br>b. Raramente.<br>c. Frecuentemente.<br>d. Siempre.
81043	VisualSymptoms	¿Con qué frecuencia se le juntan las letras al leer?	a. Nunca<br>b. Raramente.<br>c. Frecuentemente.<br>d. Siempre.
81046	VisualSymptoms	¿Con qué frecuencia ve doble al mirar de cerca?	a. Nunca<br>b. Raramente.<br>c. Frecuentemente.<br>d. Siempre.
81049	VisualSymptoms	¿Con qué frecuencia ve doble al mirar de lejos?	a. Nunca<br>b. Raramente.<br>c. Frecuentemente.<br>d. Siempre.
81052	VisualSymptoms	¿Con qué frecuencia ve peor al acabar el día?	a. Nunca<br>b. Raramente.<br>c. Frecuentemente.<br>d. Siempre.
81055	VisualSymptoms	¿Con qué frecuencia nota que ve peor al acabar de trabajar que al empezar?	a. Nunca<br>b. Raramente.<br>c. Frecuentemente.<br>d. Siempre.
81070	VisualSymptoms	¿Con qué frecuencia le cuesta cambiar la mirada de una distancia a otra?	a. Nunca<br>b. Raramente.<br>c. Frecuentemente.<br>d. Siempre.
81073	VisualSymptoms	¿Con qué frecuencia nota, al leer, que las palabras se enfocan y desenfocan?	a. Nunca<br>b. Raramente.<br>c. Frecuentemente.<br>d. Siempre.
81076	VisualSymptoms	¿Con qué frecuencia nota que le cuesta distinguir pequeños detalles?	a. Nunca<br>b. Raramente.<br>c. Frecuentemente.<br>d. Siempre.
81079	VisualSymptoms	¿Con qué frecuencia nota que veo los colores diferentes?	a. Nunca<br>b. Raramente.<br>c. Frecuentemente.<br>d. Siempre.
81082	VisualSymptoms	¿Con qué frecuencia nota que su visión fluctúa mientras trabaja?	a. Nunca<br>b. Raramente.<br>c. Frecuentemente.<br>d. Siempre.
81002	VisualSymptoms	¿Ve borroso al mirar de lejos?	a.No.<br>b. Sí, poco.<br>c. Sí, bastante.<br>d. Sí, mucho.

81005	VisualSymptoms	¿Ve borroso al mirar de cerca?	a.No.<br>b. Sí, poco.<br>c. Sí, bastante.<br>d. Sí, mucho.
81008	VisualSymptoms	¿Ve borrosa la pantalla del ordenador?	a.No.<br>b. Sí, poco.<br>c. Sí, bastante.<br>d. Sí, mucho.
81011	VisualSymptoms	¿Está satisfecho/a con su visión en cerca?	a.No.<br>b. Sí, poco.<br>c. Sí, bastante.<br>d. Sí, mucho.
81014	VisualSymptoms	¿Necesita entornar los ojos para ver bien?	a.No.<br>b. Sí, poco.<br>c. Sí, bastante.<br>d. Sí, mucho.
81017	VisualSymptoms	¿Ve peor de lo que le gustaría?	a.No.<br>b. Sí, poco.<br>c. Sí, bastante.<br>d. Sí, mucho.
81020	VisualSymptoms	¿Cree que podría ver mejor?	a.No.<br>b. Sí, poco.<br>c. Sí, bastante.<br>d. Sí, mucho.
81023	VisualSymptoms	¿Está satisfecho/a con su visión lejana?	a.No.<br>b. Sí, poco.<br>c. Sí, bastante.<br>d. Sí, mucho.
81026	VisualSymptoms	¿Le molestan las luces?	a.No.<br>b. Sí, poco.<br>c. Sí, bastante.<br>d. Sí, mucho.
81029	VisualSymptoms	¿Ve peor en ambientes poco iluminados?	a.No.<br>b. Sí, poco.<br>c. Sí, bastante.<br>d. Sí, mucho.
81032	VisualSymptoms	¿Le molestan los ojos cuando hay mucha luz?	a.No.<br>b. Sí, poco.<br>c. Sí, bastante.<br>d. Sí, mucho.
81035	VisualSymptoms	¿Ve peor en ambientes muy iluminados?	a.No.<br>b. Sí, poco.<br>c. Sí, bastante.<br>d. Sí, mucho.
81038	VisualSymptoms	¿Le cuesta ver bien cuando pasa de un ambiente muy iluminado a otro poco iluminado?	a.No.<br>b. Sí, poco.<br>c. Sí, bastante.<br>d. Sí, mucho.

81041	VisualSymptoms	¿Ve mal los letreros luminosos de la calle? Por ejemplo, el letrero de una farmacia.	a.No.<br>b. Sí, poco.<br>c. Sí, bastante.<br>d. Sí, mucho.
81044	VisualSymptoms	¿Se le juntan las letras al leer?	a.No.<br>b. Sí, poco.<br>c. Sí, bastante.<br>d. Sí, mucho.
81047	VisualSymptoms	¿Ve doble al mirar de cerca?	a.No.<br>b. Sí, poco.<br>c. Sí, bastante.<br>d. Sí, mucho.
81050	VisualSymptoms	¿Ve doble al mirar de lejos?	a.No.<br>b. Sí, poco.<br>c. Sí, bastante.<br>d. Sí, mucho.
81053	VisualSymptoms	¿Ve peor al acabar el día?	a.No.<br>b. Sí, poco.<br>c. Sí, bastante.<br>d. Sí, mucho.
81056	VisualSymptoms	¿Ve peor al acabar de trabajar que al empezar?	a.No.<br>b. Sí, poco.<br>c. Sí, bastante.<br>d. Sí, mucho.
81062	VisualSymptoms	¿Si mira por el lateral de la gafa ve peor que mirando de frente?	a.No.<br>b. Sí, poco.<br>c. Sí, bastante.<br>d. Sí, mucho.
81065	VisualSymptoms	¿Siente como si mirase a través de una lupa?	a.No.<br>b. Sí, poco.<br>c. Sí, bastante.<br>d. Sí, mucho.
81071	VisualSymptoms	¿Le cuesta enfocar cuando cambia la mirada de una distancia a otra?	a.No.<br>b. Sí, poco.<br>c. Sí, bastante.<br>d. Sí, mucho.
81074	VisualSymptoms	¿Cuando lee nota que las palabras se enfocan y desenfocan?	a.No.<br>b. Sí, poco.<br>c. Sí, bastante.<br>d. Sí, mucho.
81077	VisualSymptoms	¿Nota que le cuesta distinguir pequeños detalles?	a.No.<br>b. Sí, poco.<br>c. Sí, bastante.<br>d. Sí, mucho.
81080	VisualSymptoms	¿Ve los colores diferentes?	a.No.<br>b. Sí, poco.<br>c. Sí, bastante.<br>d. Sí, mucho.

		¿Cómo de cierta es la siguiente afirmación para usted? Usando gafas...	
81003	VisualSymptoms	Usando gafas, veo mal de lejos.	a. Totalmente falsa.<br>b. Bastante falsa.<br>c. Bastante cierto.<br>d. Totalmente cierto.
81006	VisualSymptoms	Usando gafas, veo mal de cerca.	a. Totalmente falsa.<br>b. Bastante falsa.<br>c. Bastante cierto.<br>d. Totalmente cierto.
81009	VisualSymptoms	Usando gafas, veo borrosa la pantalla del ordenador.	a. Totalmente falsa.<br>b. Bastante falsa.<br>c. Bastante cierto.<br>d. Totalmente cierto.
81012	VisualSymptoms	Estoy satisfecho/a con mi visión de cerca, usando gafas.	a. Totalmente falsa.<br>b. Bastante falsa.<br>c. Bastante cierto.<br>d. Totalmente cierto.
81015	VisualSymptoms	Con mis gafas puestas, entorno los ojos para ver bien.	a. Totalmente falsa.<br>b. Bastante falsa.<br>c. Bastante cierto.<br>d. Totalmente cierto.
81018	VisualSymptoms	Con mis gafas, veo peor de lo que me gustaría.	a. Totalmente falsa.<br>b. Bastante falsa.<br>c. Bastante cierto.<br>d. Totalmente cierto.
81021	VisualSymptoms	Me gustaría ver mejor con mis gafas.	a. Totalmente falsa.<br>b. Bastante falsa.<br>c. Bastante cierto.<br>d. Totalmente cierto.
81024	VisualSymptoms	Estoy satisfecho/a con mi visión de lejos, usando gafas.	a. Totalmente falsa.<br>b. Bastante falsa.<br>c. Bastante cierto.<br>d. Totalmente cierto.
81027	VisualSymptoms	Llevando gafas, me molestan las luces.	a. Totalmente falsa.<br>b. Bastante falsa.<br>c. Bastante cierto.<br>d. Totalmente cierto.
81030	VisualSymptoms	Usando gafas, veo peor en ambientes poco iluminados.	a. Totalmente falsa.<br>b. Bastante falsa.<br>c. Bastante cierto.<br>d. Totalmente cierto.
81033	VisualSymptoms	Llevando gafas, me molestan los ojos cuando hay mucha luz.	a. Totalmente falsa.<br>b. Bastante falsa.<br>c. Bastante cierto.<br>d. Totalmente cierto.

81036	VisualSymptoms	Veo peor en ambientes muy iluminados, usando gafas.	a. Totalmente falsa.<br>b. Bastante falsa.<br>c. Bastante cierto.<br>d. Totalmente cierto.
81039	VisualSymptoms	Con mis gafas, me cuesta cambiar de un ambiente muy iluminado a otro poco iluminado.	a. Totalmente falsa.<br>b. Bastante falsa.<br>c. Bastante cierto.<br>d. Totalmente cierto.
81042	VisualSymptoms	Llevando gafas, veo mal los letreros luminosos de la calle.	a. Totalmente falsa.<br>b. Bastante falsa.<br>c. Bastante cierto.<br>d. Totalmente cierto.
81045	VisualSymptoms	Al leer con mis gafas, las letras se juntan.	a. Totalmente falsa.<br>b. Bastante falsa.<br>c. Bastante cierto.<br>d. Totalmente cierto.
81048	VisualSymptoms	Usando gafas, veo doble al mirar de cerca.	a. Totalmente falsa.<br>b. Bastante falsa.<br>c. Bastante cierto.<br>d. Totalmente cierto.
81051	VisualSymptoms	Veo doble al mirar de lejos con mis gafas.	a. Totalmente falsa.<br>b. Bastante falsa.<br>c. Bastante cierto.<br>d. Totalmente cierto.
81054	VisualSymptoms	Veo peor al acabar el día.	a. Totalmente falsa.<br>b. Bastante falsa.<br>c. Bastante cierto.<br>d. Totalmente cierto.
81057	VisualSymptoms	Veo peor al acabar de trabajar que al empezar.	a. Totalmente falsa.<br>b. Bastante falsa.<br>c. Bastante cierto.<br>d. Totalmente cierto.
81063	VisualSymptoms	Veo peor al mirar de lado por las gafas que mirando de frente.	a. Totalmente falsa.<br>b. Bastante falsa.<br>c. Bastante cierto.<br>d. Totalmente cierto.
81066	VisualSymptoms	Cuando uso mis gafas, siento que miro a través de una lupa.	a. Totalmente falsa.<br>b. Bastante falsa.<br>c. Bastante cierto.<br>d. Totalmente cierto.
81072	VisualSymptoms	Con mis gafas, me cuesta enfocar al cambiar la mirada de una distancia a otra.	a. Totalmente falsa.<br>b. Bastante falsa.<br>c. Bastante cierto.<br>d. Totalmente cierto.
81075	VisualSymptoms	Al leer con mis gafas, las palabras se enfocan y desenfocan.	a. Totalmente falsa.<br>b. Bastante falsa.<br>c. Bastante cierto.<br>d. Totalmente cierto.

81078	VisualSymptoms	Me cuesta distinguir detalles pequeños.	a. Totalmente falsa.<br>b. Bastante falsa.<br>c. Bastante cierto.<br>d. Totalmente cierto.
81081	VisualSymptoms	Veo los colores diferentes.	a. Totalmente falsa.<br>b. Bastante falsa.<br>c. Bastante cierto.<br>d. Totalmente cierto.
81083	VisualSymptoms	Mi visión fluctúa mientras trabajo.	a. Totalmente falsa.<br>b. Bastante falsa.<br>c. Bastante cierto.<br>d. Totalmente cierto.
id	trait	Usando lentillas:	
83001	VisualSymptoms	¿Con qué frecuencia ve borroso al mirar de lejos?	a. Nunca<br>b. Raramente.<br>c. Frecuentemente.<br>d. Siempre.
83004	VisualSymptoms	¿Con qué frecuencia ve borroso al mirar de cerca?	a. Nunca<br>b. Raramente.<br>c. Frecuentemente.<br>d. Siempre.
83007	VisualSymptoms	¿Con qué frecuencia ve borrosa la pantalla del ordenador?	a. Nunca<br>b. Raramente.<br>c. Frecuentemente.<br>d. Siempre.
83013	VisualSymptoms	¿Con qué frecuencia necesita entornar los ojos para ver bien?	a. Nunca<br>b. Raramente.<br>c. Frecuentemente.<br>d. Siempre.
83016	VisualSymptoms	¿Con qué frecuencia ve peor de lo que le gustaría?	a. Nunca<br>b. Raramente.<br>c. Frecuentemente.<br>d. Siempre.
83025	VisualSymptoms	¿Con qué frecuencia le molestan las luces?	a. Nunca<br>b. Raramente.<br>c. Frecuentemente.<br>d. Siempre.
83028	VisualSymptoms	¿Con qué frecuencia ve peor en ambientes poco iluminados?	a. Nunca<br>b. Raramente.<br>c. Frecuentemente.<br>d. Siempre.
83031	VisualSymptoms	¿Con qué frecuencia le molestan los ojos cuando hay mucha luz?	a. Nunca<br>b. Raramente.<br>c. Frecuentemente.<br>d. Siempre.

83034	VisualSymptoms	¿Con qué frecuencia ve peor en ambientes muy iluminados?	a. Nunca<br>b. Raramente.<br>c. Frecuentemente.<br>d. Siempre.
83037	VisualSymptoms	¿Con qué frecuencia le cuesta ver bien, cuando pasa de un ambiente muy iluminado a otro poco iluminado?	a. Nunca<br>b. Raramente.<br>c. Frecuentemente.<br>d. Siempre.
83040	VisualSymptoms	¿Con qué frecuencia ve mal los letreros luminosos de la calle? Por ejemplo, el letrero de una farmacia.	a. Nunca<br>b. Raramente.<br>c. Frecuentemente.<br>d. Siempre.
83043	VisualSymptoms	¿Con qué frecuencia se le juntan las letras al leer?	a. Nunca<br>b. Raramente.<br>c. Frecuentemente.<br>d. Siempre.
83046	VisualSymptoms	¿Con qué frecuencia ve doble al mirar de cerca?	a. Nunca<br>b. Raramente.<br>c. Frecuentemente.<br>d. Siempre.
83049	VisualSymptoms	¿Con qué frecuencia ve doble al mirar de lejos?	a. Nunca<br>b. Raramente.<br>c. Frecuentemente.<br>d. Siempre.
83052	VisualSymptoms	¿Con qué frecuencia ve peor al acabar el día?	a. Nunca<br>b. Raramente.<br>c. Frecuentemente.<br>d. Siempre.
83055	VisualSymptoms	¿Con qué frecuencia nota que ve peor al acabar de trabajar que al empezar?	a. Nunca<br>b. Raramente.<br>c. Frecuentemente.<br>d. Siempre.
83070	VisualSymptoms	¿Con qué frecuencia nota que le cuesta cambiar la mirada de una distancia a otra?	a. Nunca<br>b. Raramente.<br>c. Frecuentemente.<br>d. Siempre.
83073	VisualSymptoms	¿Con qué frecuencia nota al leer que las palabras se enfocan y desenfocan?	a. Nunca<br>b. Raramente.<br>c. Frecuentemente.<br>d. Siempre.
83076	VisualSymptoms	¿Con qué frecuencia nota que le cuesta distinguir pequeños detalles?	a. Nunca<br>b. Raramente.<br>c. Frecuentemente.<br>d. Siempre.
83079	VisualSymptoms	¿Con qué frecuencia nota que veo los colores diferentes?	a. Nunca<br>b. Raramente.<br>c. Frecuentemente.<br>d. Siempre.

83082	VisualSymptoms	¿Con qué frecuencia, mientras lee, ve sombras tras las letras?	a. Nunca<br>b. Raramente.<br>c. Frecuentemente.<br>d. Siempre.
83085	VisualSymptoms	¿Con qué frecuencia nota que su visión fluctúa mientras trabaja?	a. Nunca<br>b. Raramente.<br>c. Frecuentemente.<br>d. Siempre.
83002	VisualSymptoms	¿Ve borroso al mirar de lejos?	a.No.<br>b. Sí, poco.<br>c. Sí, bastante.<br>d. Sí, mucho.
83005	VisualSymptoms	¿Ve borroso al mirar de cerca?	a.No.<br>b. Sí, poco.<br>c. Sí, bastante.<br>d. Sí, mucho.
83008	VisualSymptoms	¿Ve borrosa la pantalla del ordenador?	a.No.<br>b. Sí, poco.<br>c. Sí, bastante.<br>d. Sí, mucho.
83011	VisualSymptoms	¿Está satisfecho/a con su visión en cerca?	a.No.<br>b. Sí, poco.<br>c. Sí, bastante.<br>d. Sí, mucho.
83014	VisualSymptoms	¿Necesita entornar los ojos para ver bien?	a.No.<br>b. Sí, poco.<br>c. Sí, bastante.<br>d. Sí, mucho.
83017	VisualSymptoms	¿Ve peor de lo que le gustaría?	a.No.<br>b. Sí, poco.<br>c. Sí, bastante.<br>d. Sí, mucho.
83020	VisualSymptoms	¿Piensa que podría ver mejor?	a.No.<br>b. Sí, poco.<br>c. Sí, bastante.<br>d. Sí, mucho.
83023	VisualSymptoms	¿Está satisfecho/a con su visión lejana?	a.No.<br>b. Sí, poco.<br>c. Sí, bastante.<br>d. Sí, mucho.
83026	VisualSymptoms	¿Le molestan las luces?	a.No.<br>b. Sí, poco.<br>c. Sí, bastante.<br>d. Sí, mucho.
83029	VisualSymptoms	¿Ve peor en ambientes poco iluminados?	a.No.<br>b. Sí, poco.<br>c. Sí, bastante.<br>d. Sí, mucho.

83032	VisualSymptoms	¿Le molestan los ojos cuando hay mucha luz?	a.No.<br>b. Sí, poco.<br>c. Sí, bastante.<br>d. Sí, mucho.
83035	VisualSymptoms	¿Ve peor en ambientes muy iluminados?	a.No.<br>b. Sí, poco.<br>c. Sí, bastante.<br>d. Sí, mucho.
83038	VisualSymptoms	¿Le cuesta ver bien cuando pasa de un ambiente muy iluminado a otro poco iluminado?	a.No.<br>b. Sí, poco.<br>c. Sí, bastante.<br>d. Sí, mucho.
83041	VisualSymptoms	¿Ve mal los letreros luminosos de la calle? Por ejemplo, el letrero de una farmacia.	a.No.<br>b. Sí, poco.<br>c. Sí, bastante.<br>d. Sí, mucho.
83044	VisualSymptoms	¿Se le juntan las letras al leer?	a.No.<br>b. Sí, poco.<br>c. Sí, bastante.<br>d. Sí, mucho.
83047	VisualSymptoms	¿Ve doble al mirar de cerca?	a.No.<br>b. Sí, poco.<br>c. Sí, bastante.<br>d. Sí, mucho.
83050	VisualSymptoms	¿Ve doble al mirar de lejos?	a.No.<br>b. Sí, poco.<br>c. Sí, bastante.<br>d. Sí, mucho.
83053	VisualSymptoms	¿Ve peor al acabar el día?	a.No.<br>b. Sí, poco.<br>c. Sí, bastante.<br>d. Sí, mucho.
83056	VisualSymptoms	¿Ve peor al acabar de trabajar que al empezar?	a.No.<br>b. Sí, poco.<br>c. Sí, bastante.<br>d. Sí, mucho.
83059	VisualSymptoms	¿Ve las letras con sombra?	a.No.<br>b. Sí, poco.<br>c. Sí, bastante.<br>d. Sí, mucho.
83062	VisualSymptoms	¿Nota al leer que las palabras se enfocan y desenfocan?	a.No.<br>b. Sí, poco.<br>c. Sí, bastante.<br>d. Sí, mucho.
83068	VisualSymptoms	¿Ve peor de cerca cuando usa lentillas?	a.No.<br>b. Sí, poco.<br>c. Sí, bastante.<br>d. Sí, mucho.

83071	VisualSymptoms	¿Le cuesta cambiar la mirada de una distancia a otra?	a.No.<br>b. Sí, poco.<br>c. Sí, bastante.<br>d. Sí, mucho.
83074	VisualSymptoms	¿Empeora su visión al usar lentillas?	a.No.<br>b. Sí, poco.<br>c. Sí, bastante.<br>d. Sí, mucho.
83077	VisualSymptoms	¿Nota que le cuesta distinguir pequeños detalles?	a.No.<br>b. Sí, poco.<br>c. Sí, bastante.<br>d. Sí, mucho.
83080	VisualSymptoms	¿Ve los colores diferentes?	a.No.<br>b. Sí, poco.<br>c. Sí, bastante.<br>d. Sí, mucho.
83083	VisualSymptoms	Leyendo ¿Ve sombras tras las letras?	a.No.<br>b. Sí, poco.<br>c. Sí, bastante.<br>d. Sí, mucho.
83086	VisualSymptoms	¿Su visión fluctúa mientras trabaja?	a.No.<br>b. Sí, poco.<br>c. Sí, bastante.<br>d. Sí, mucho.
		¿Cómo de cierta es la siguiente afirmación para usted? Usando lentillas...	
83003	VisualSymptoms	Con mis lentillas, veo mal de lejos.	a. Totalmente falsa.<br>b. Bastante falsa.<br>c. Bastante cierto.<br>d. Totalmente cierto.
83006	VisualSymptoms	Con mis lentillas, veo mal de cerca.	a. Totalmente falsa.<br>b. Bastante falsa.<br>c. Bastante cierto.<br>d. Totalmente cierto.
83009	VisualSymptoms	Con lentillas veo borrosa la pantalla del ordenador.	a. Totalmente falsa.<br>b. Bastante falsa.<br>c. Bastante cierto.<br>d. Totalmente cierto.
83012	VisualSymptoms	Estoy satisfecho/a con mi visión de cerca, usando lentillas.	a. Totalmente falsa.<br>b. Bastante falsa.<br>c. Bastante cierto.<br>d. Totalmente cierto.
83015	VisualSymptoms	Usando lentillas, tengo que entornar los ojos para ver bien.	a. Totalmente falsa.<br>b. Bastante falsa.<br>c. Bastante cierto.<br>d. Totalmente cierto.

83018	VisualSymptoms	Veo peor de lo que me gustaría, con mis lentillas.	a. Totalmente falsa.<br>b. Bastante falsa.<br>c. Bastante cierto.<br>d. Totalmente cierto.
83021	VisualSymptoms	Me gustaría ver mejor con mis lentillas.	a. Totalmente falsa.<br>b. Bastante falsa.<br>c. Bastante cierto.<br>d. Totalmente cierto.
83024	VisualSymptoms	Estoy satisfecho/a con mi visión de lejos, usando lentillas.	a. Totalmente falsa.<br>b. Bastante falsa.<br>c. Bastante cierto.<br>d. Totalmente cierto.
83027	VisualSymptoms	Usando lentillas, me molestan las luces.	a. Totalmente falsa.<br>b. Bastante falsa.<br>c. Bastante cierto.<br>d. Totalmente cierto.
83030	VisualSymptoms	Con lentillas, veo peor en ambientes poco iluminados.	a. Totalmente falsa.<br>b. Bastante falsa.<br>c. Bastante cierto.<br>d. Totalmente cierto.
83033	VisualSymptoms	Usando lentillas, me molestan los ojos si hay mucha luz.	a. Totalmente falsa.<br>b. Bastante falsa.<br>c. Bastante cierto.<br>d. Totalmente cierto.
83036	VisualSymptoms	Con lentillas, veo peor en ambientes muy iluminados.	a. Totalmente falsa.<br>b. Bastante falsa.<br>c. Bastante cierto.<br>d. Totalmente cierto.
83039	VisualSymptoms	Usando lentillas, me cuesta ver bien al cambiar de un ambiente muy iluminado a otro poco iluminado.	a. Totalmente falsa.<br>b. Bastante falsa.<br>c. Bastante cierto.<br>d. Totalmente cierto.
83042	VisualSymptoms	Con las lentillas, veo mal los letreros luminosos de la calle.	a. Totalmente falsa.<br>b. Bastante falsa.<br>c. Bastante cierto.<br>d. Totalmente cierto.
83045	VisualSymptoms	Cuando leo con lentillas, noto que las letras se juntan.	a. Totalmente falsa.<br>b. Bastante falsa.<br>c. Bastante cierto.<br>d. Totalmente cierto.
83048	VisualSymptoms	Usando mis lentillas, veo doble al mirar de cerca.	a. Totalmente falsa.<br>b. Bastante falsa.<br>c. Bastante cierto.<br>d. Totalmente cierto.
83051	VisualSymptoms	Usando mis lentillas, veo doble al mirar de lejos.	a. Totalmente falsa.<br>b. Bastante falsa.<br>c. Bastante cierto.<br>d. Totalmente cierto.

83054	VisualSymptoms	Veo peor al acabar el día.	a. Totalmente falsa.<br>b. Bastante falsa.<br>c. Bastante cierto.<br>d. Totalmente cierto.
83057	VisualSymptoms	Veo peor al acabar de trabajar que al empezar.	a. Totalmente falsa.<br>b. Bastante falsa.<br>c. Bastante cierto.<br>d. Totalmente cierto.
83060	VisualSymptoms	Usando mis lentillas, veo las letras con sombras.	a. Totalmente falsa.<br>b. Bastante falsa.<br>c. Bastante cierto.<br>d. Totalmente cierto.
83063	VisualSymptoms	Al leer, usando mis lentillas, las palabras se enfocan y desenfocan.	a. Totalmente falsa.<br>b. Bastante falsa.<br>c. Bastante cierto.<br>d. Totalmente cierto.
83072	VisualSymptoms	Usando lentillas, me resulta difícil cambiar la mirada de una distancia a otra.	a. Totalmente falsa.<br>b. Bastante falsa.<br>c. Bastante cierto.<br>d. Totalmente cierto.
84076	VisualSymptoms	Veo peor con mis lentillas que con mis gafas.	a. Totalmente falsa.<br>b. Bastante falsa.<br>c. Bastante cierto.<br>d. Totalmente cierto.
84077	VisualSymptoms	De cerca, veo peor con mis lentillas que con mis gafas.	a. Totalmente falsa.<br>b. Bastante falsa.<br>c. Bastante cierto.<br>d. Totalmente cierto.
84078	VisualSymptoms	De lejos, veo peor con mis lentillas que con mis gafas.	a. Totalmente falsa.<br>b. Bastante falsa.<br>c. Bastante cierto.<br>d. Totalmente cierto.
83075	VisualSymptoms	Me cuesta distinguir detalles pequeños.	a. Totalmente falsa.<br>b. Bastante falsa.<br>c. Bastante cierto.<br>d. Totalmente cierto.
83078	VisualSymptoms	Veo los colores diferentes.	a. Totalmente falsa.<br>b. Bastante falsa.<br>c. Bastante cierto.<br>d. Totalmente cierto.
83081	VisualSymptoms	Leyendo con lentillas veo sobras tras las letras.	a. Totalmente falsa.<br>b. Bastante falsa.<br>c. Bastante cierto.<br>d. Totalmente cierto.
83084	VisualSymptoms	Mi visión fluctúa mientras trabajo.	a. Totalmente falsa.<br>b. Bastante falsa.<br>c. Bastante cierto.<br>d. Totalmente cierto.

id	trait	Sin usar gafas o lentillas:	
82001	VisualSymptoms	¿Con qué frecuencia ve borroso al mirar de lejos?	a. Nunca<br>b. Raramente.<br>c. Frecuentemente.<br>d. Siempre.
82004	VisualSymptoms	¿Con qué frecuencia ve borroso al mirar de cerca?	a. Nunca<br>b. Raramente.<br>c. Frecuentemente.<br>d. Siempre.
82007	VisualSymptoms	¿Con qué frecuencia ve borrosa la pantalla del ordenador?	a. Nunca<br>b. Raramente.<br>c. Frecuentemente.<br>d. Siempre.
82013	VisualSymptoms	¿Con qué frecuencia necesita entornar los ojos para ver bien?	a. Nunca<br>b. Raramente.<br>c. Frecuentemente.<br>d. Siempre.
82016	VisualSymptoms	¿Con qué frecuencia ve peor de lo que le gustaría?	a. Nunca<br>b. Raramente.<br>c. Frecuentemente.<br>d. Siempre.
82019	VisualSymptoms	¿Con qué frecuencia cree que podría ver mejor?	a. Nunca<br>b. Raramente.<br>c. Frecuentemente.<br>d. Siempre.
82025	VisualSymptoms	¿Con qué frecuencia le molestan las luces?	a. Nunca<br>b. Raramente.<br>c. Frecuentemente.<br>d. Siempre.
82028	VisualSymptoms	¿Con qué frecuencia ve peor en ambientes poco iluminados?	a. Nunca<br>b. Raramente.<br>c. Frecuentemente.<br>d. Siempre.
82031	VisualSymptoms	¿Con qué frecuencia le molestan los ojos cuando hay mucha luz?	a. Nunca<br>b. Raramente.<br>c. Frecuentemente.<br>d. Siempre.
82034	VisualSymptoms	¿Con qué frecuencia ve peor en ambientes muy iluminados?	a. Nunca<br>b. Raramente.<br>c. Frecuentemente.<br>d. Siempre.
82037	VisualSymptoms	¿Con qué frecuencia le cuesta ver bien, cuando pasa de un ambiente muy iluminado a otro poco iluminado?	a. Nunca<br>b. Raramente.<br>c. Frecuentemente.<br>d. Siempre.

82040	VisualSymptoms	¿Con qué frecuencia ve mal los letreros luminosos de la calle? Por ejemplo, el letrero de una farmacia.	a. Nunca<br>b. Raramente.<br>c. Frecuentemente.<br>d. Siempre.
82043	VisualSymptoms	¿Con qué frecuencia se le juntan las letras al leer?	a. Nunca<br>b. Raramente.<br>c. Frecuentemente.<br>d. Siempre.
82046	VisualSymptoms	¿Con qué frecuencia ve doble al mirar de cerca?	a. Nunca<br>b. Raramente.<br>c. Frecuentemente.<br>d. Siempre.
82049	VisualSymptoms	¿Con qué frecuencia ve doble al mirar de lejos?	a. Nunca<br>b. Raramente.<br>c. Frecuentemente.<br>d. Siempre.
82052	VisualSymptoms	¿Con qué frecuencia ve peor al acabar el día?	a. Nunca<br>b. Raramente.<br>c. Frecuentemente.<br>d. Siempre.
82055	VisualSymptoms	¿Con qué frecuencia nota que ve peor al acabar de trabajar que al empezar?	a. Nunca<br>b. Raramente.<br>c. Frecuentemente.<br>d. Siempre.
82070	VisualSymptoms	¿Con qué frecuencia nota que le cuesta cambiar la mirada de una distancia a otra?	a. Nunca<br>b. Raramente.<br>c. Frecuentemente.<br>d. Siempre.
82073	VisualSymptoms	¿Con qué frecuencia nota al leer que las palabras se enfocan y desenfocan?	a. Nunca<br>b. Raramente.<br>c. Frecuentemente.<br>d. Siempre.
83076	VisualSymptoms	¿Con qué frecuencia nota que le cuesta distinguir pequeños detalles?	a. Nunca<br>b. Raramente.<br>c. Frecuentemente.<br>d. Siempre.
82002	VisualSymptoms	¿Ve borroso al mirar de lejos?	a.No.<br>b. Sí, poco.<br>c. Sí, bastante.<br>d. Sí, mucho.
82005	VisualSymptoms	¿Ve borroso al mirar de cerca?	a.No.<br>b. Sí, poco.<br>c. Sí, bastante.<br>d. Sí, mucho.
82008	VisualSymptoms	¿Ve borrosa la pantalla del ordenador?	a.No.<br>b. Sí, poco.<br>c. Sí, bastante.<br>d. Sí, mucho.

82011	VisualSymptoms	¿Está satisfecho/a con su visión en cerca?	a.No.<br>b. Sí, poco.<br>c. Sí, bastante.<br>d. Sí, mucho.
82014	VisualSymptoms	¿Necesita entornar los ojos para ver bien?	a.No.<br>b. Sí, poco.<br>c. Sí, bastante.<br>d. Sí, mucho.
82017	VisualSymptoms	¿Ve peor de lo que le gustaría?	a.No.<br>b. Sí, poco.<br>c. Sí, bastante.<br>d. Sí, mucho.
82020	VisualSymptoms	¿Piensa que podría ver mejor?	a.No.<br>b. Sí, poco.<br>c. Sí, bastante.<br>d. Sí, mucho.
82023	VisualSymptoms	¿Está satisfecho/a con su visión lejana?	a.No.<br>b. Sí, poco.<br>c. Sí, bastante.<br>d. Sí, mucho.
82026	VisualSymptoms	¿Le molestan las luces?	a.No.<br>b. Sí, poco.<br>c. Sí, bastante.<br>d. Sí, mucho.
82029	VisualSymptoms	¿Ve peor en ambientes poco iluminados?	a.No.<br>b. Sí, poco.<br>c. Sí, bastante.<br>d. Sí, mucho.
82032	VisualSymptoms	¿Le molestan los ojos cuando hay mucha luz?	a.No.<br>b. Sí, poco.<br>c. Sí, bastante.<br>d. Sí, mucho.
82035	VisualSymptoms	¿Ve peor en ambientes muy iluminados?	a.No.<br>b. Sí, poco.<br>c. Sí, bastante.<br>d. Sí, mucho.
82038	VisualSymptoms	¿Le cuesta ver bien cuando pasa de un ambiente muy iluminado a otro poco iluminado?	a.No.<br>b. Sí, poco.<br>c. Sí, bastante.<br>d. Sí, mucho.
82041	VisualSymptoms	¿Ve mal los letreros luminosos de la calle? Por ejemplo, el letrero de una farmacia.	a.No.<br>b. Sí, poco.<br>c. Sí, bastante.<br>d. Sí, mucho.
82044	VisualSymptoms	¿Se le juntan las letras al leer?	a.No.<br>b. Sí, poco.<br>c. Sí, bastante.<br>d. Sí, mucho.

82047	VisualSymptoms	¿Ve doble al mirar de cerca?	a.No.<br>b. Sí, poco.<br>c. Sí, bastante.<br>d. Sí, mucho.
82050	VisualSymptoms	¿Ve doble al mirar de lejos?	a.No.<br>b. Sí, poco.<br>c. Sí, bastante.<br>d. Sí, mucho.
82053	VisualSymptoms	¿Ve peor al acabar el día?	a.No.<br>b. Sí, poco.<br>c. Sí, bastante.<br>d. Sí, mucho.
82056	VisualSymptoms	¿Ve peor al acabar de trabajar que al empezar?	a.No.<br>b. Sí, poco.<br>c. Sí, bastante.<br>d. Sí, mucho.
82062	VisualSymptoms	¿Nota al leer que las palabras se enfocan y desenfocan?	a.No.<br>b. Sí, poco.<br>c. Sí, bastante.<br>d. Sí, mucho.
82071	VisualSymptoms	¿Le cuesta cambiar la mirada de una distancia a otra?	a.No.<br>b. Sí, poco.<br>c. Sí, bastante.<br>d. Sí, mucho.
82074	VisualSymptoms	¿Nota que le cuesta distinguir pequeños detalles?	a.No.<br>b. Sí, poco.<br>c. Sí, bastante.<br>d. Sí, mucho.
		¿Cómo de cierta es la siguiente afirmación para usted? Sin usar gafas ni lentillas...	
82003	VisualSymptoms	Veo mal de lejos.	a. Totalmente falsa.<br>b. Bastante falsa.<br>c. Bastante cierto.<br>d. Totalmente cierto.
82006	VisualSymptoms	Veo mal de cerca.	a. Totalmente falsa.<br>b. Bastante falsa.<br>c. Bastante cierto.<br>d. Totalmente cierto.
82009	VisualSymptoms	Veo borrosa la pantalla del ordenador.	a. Totalmente falsa.<br>b. Bastante falsa.<br>c. Bastante cierto.<br>d. Totalmente cierto.
82012	VisualSymptoms	Estoy satisfecho/a con mi visión de cerca.	a. Totalmente falsa.<br>b. Bastante falsa.<br>c. Bastante cierto.<br>d. Totalmente cierto.

82015	VisualSymptoms	Necesito entornar los ojos para ver bien.	a. Totalmente falsa.<br>b. Bastante falsa.<br>c. Bastante cierto.<br>d. Totalmente cierto.
82018	VisualSymptoms	Veo peor de lo que me gustaría.	a. Totalmente falsa.<br>b. Bastante falsa.<br>c. Bastante cierto.<br>d. Totalmente cierto.
82024	VisualSymptoms	Estoy satisfecho/a con mi visión de lejos.	a. Totalmente falsa.<br>b. Bastante falsa.<br>c. Bastante cierto.<br>d. Totalmente cierto.
82027	VisualSymptoms	Me molestan las luces.	a. Totalmente falsa.<br>b. Bastante falsa.<br>c. Bastante cierto.<br>d. Totalmente cierto.
82030	VisualSymptoms	Veo peor en ambientes poco iluminados.	a. Totalmente falsa.<br>b. Bastante falsa.<br>c. Bastante cierto.<br>d. Totalmente cierto.
82033	VisualSymptoms	Me molestan los ojos si hay mucha luz.	a. Totalmente falsa.<br>b. Bastante falsa.<br>c. Bastante cierto.<br>d. Totalmente cierto.
82036	VisualSymptoms	Veo peor en ambientes muy iluminados.	a. Totalmente falsa.<br>b. Bastante falsa.<br>c. Bastante cierto.<br>d. Totalmente cierto.
82039	VisualSymptoms	Me cuesta ver bien al cambiar de un ambiente muy iluminado a otro poco iluminado.	a. Totalmente falsa.<br>b. Bastante falsa.<br>c. Bastante cierto.<br>d. Totalmente cierto.
82042	VisualSymptoms	Veo mal los letreros luminosos de la calle.	a. Totalmente falsa.<br>b. Bastante falsa.<br>c. Bastante cierto.<br>d. Totalmente cierto.
82045	VisualSymptoms	Noto que las letras se juntan.	a. Totalmente falsa.<br>b. Bastante falsa.<br>c. Bastante cierto.<br>d. Totalmente cierto.
82048	VisualSymptoms	Veo doble al mirar de cerca.	a. Totalmente falsa.<br>b. Bastante falsa.<br>c. Bastante cierto.<br>d. Totalmente cierto.
82051	VisualSymptoms	Veo doble al mirar de lejos.	a. Totalmente falsa.<br>b. Bastante falsa.<br>c. Bastante cierto.<br>d. Totalmente cierto.

82054	VisualSymptoms	Veo peor al acabar el día.	a. Totalmente falsa.<br>b. Bastante falsa.<br>c. Bastante cierto.<br>d. Totalmente cierto.
82057	VisualSymptoms	Veo peor al acabar de trabajar que al empezar.	a. Totalmente falsa.<br>b. Bastante falsa.<br>c. Bastante cierto.<br>d. Totalmente cierto.
82063	VisualSymptoms	Al leer las palabras se enfocan y desenfocan.	a. Totalmente falsa.<br>b. Bastante falsa.<br>c. Bastante cierto.<br>d. Totalmente cierto.
82072	VisualSymptoms	Me resulta difícil cambiar la mirada de una distancia a otra.	a. Totalmente falsa.<br>b. Bastante falsa.<br>c. Bastante cierto.<br>d. Totalmente cierto.
82075	VisualSymptoms	Me cuesta distinguir detalles pequeños.	a. Totalmente falsa.<br>b. Bastante falsa.<br>c. Bastante cierto.<br>d. Totalmente cierto.
