# Supplementary material for: Content development for a new item-bank for measuring multifocal contact lens performance
Source: J Patient Rep Outcomes. 2024 Feb 8;8:16. doi: 10.1186/s41687-024-00689-w (PMC10853121; doi:10.1186/s41687-024-00689-w)
Supplement: Supplementary file 2 — Additional File 2: Focus Group Guide. This document contains the guide that the moderator followed during the focus groups. The content is in Spanish since the focus groups were conducted in this language [file 41687_2024_689_MOESM2_ESM.pdf]

Guía para “focus groups”

Paciente:

1. Presentación:
- Nombre, Edad, Profesión...
- ¿A que dedica su tiempo libre?
- ¿Cuando empezó a sufrir vista cansada?

2. ¿Cómo definiría la presbicia o vista cansada?
3. Preguntas apoyo:
- Cosas que más te molestan de llevar gafas
- Cosas que más te molestan del uso de lentes de contacto (lentillas)
- Primeros síntomas que notaste de vista cansada
- Situaciones incomodas a raíz de padecer vista cansada
- Actividades de tu día a día en las que te ves limitado por la presbicia
- Consecuencias de tener vista cansada
- Nuevas responsabilidades que has adquirido desde que tienes vista cansada
- Soluciones que buscaste cuando comenzaste a notar problemas de visión
- Molestias visuales que se presentan aun habiendo corregido el problema

Preguntar directamente sobre temas economicos ya que no suelen salir en la conversación.

Profesionales o Profesores

El objetivo es que la conversación sea fluida y todos participen. Para esto, podemos empezar con una breve presentación, (nombre y especialidad o campo de trabajo). A continuación, podemos utilizar las siguientes preguntas con el fin de orientar la discusión:

1. ¿Qué motivos consideraríais los más populares por los cuales un paciente tiene éxito en su adaptación de LC?
2. ¿Encuentran los pacientes présbitas mayor dificultad en la adaptación de LC progresivas? ¿Se observa una relación entre pacientes que ya han sido usuarios de LC y su éxito en la adaptación de las progresivas? ¿O son estos más exigentes en cuanto a calidad visual?
3. ¿Muestran los pacientes présbitas mayor sensibilidad a la hora de utilizar LC? ¿Encuentran más molestias debido a la edad?
4. ¿Cuáles son las actividades diarias que los pacientes manifiestan como un mayor reto mientras usan LC progresivas?
5. Cuando recibimos a un paciente présbita ¿Cuáles son las circunstancias que nos hace ofrecerle la solución de las LC progresivas? ¿Cuáles son a vuestro parecer las ventajas de este método?

6. ¿Cuáles consideráis los mayores retos de las LC progresivas? diseño óptico, material… ¿Qué hace que elijamos una u otra para la adaptación?
7. ¿Qué molestias presentan los pacientes más a menudo? ¿Qué les aconsejamos frente a esta situación?
8. ¿Consideráis que los pacientes présbitas consiguen llegar al final de la adaptación? ¿Es el tiempo de adaptación un problema en abandono de LC?
9. ¿Cómo pensáis que el desconocimiento de la estructura ocular y el método de corrección utilizado es un obstáculo para el paciente?
10. ¿Es el precio un obstáculo para los présbitas a la hora de probar nuevos métodos de corrección? ¿hay un factor económico asociado a la salud visual?
