# Supplementary material for: Content development for a new item-bank for measuring multifocal contact lens performance
Source: J Patient Rep Outcomes. 2024 Feb 8;8:16. doi: 10.1186/s41687-024-00689-w (PMC10853121; doi:10.1186/s41687-024-00689-w)
Supplement: Supplementary file 3 — Additional File 3: Item construction information. This file is compose of 2 tables. This material did not undergo formal translation from Spanish to English, therefore a cross-cultural validation process should be performed before it is used. Table 1: presents examples of items included in the item bank. Table 2: shows quotes extracted from the different research methods supporting an item. This table should be read horizontally [file 41687_2024_689_MOESM3_ESM.pdf]

Table 1.

Domain:	Question format:	Answering format:
**Cognitive issues**	To what extent is the following statement true? *…*<br>*E.g.* To what extent is the following statement true? *I forget where I leave my things.*	*a.* Not true at all.<br>*b.* Somewhat true.<br>*c.* Mostly true.<br>*d.* Completely true
**Economic Issues**	*Not structured.*<br>*E.g. Do you consider that the benefits of progressive contact lenses justify their cost?*<br>*a. Not at all.*<br>*b. Somewhat.*<br>*c. Mostly.*<br>*d. Completely.*	
**Emotional Well-Being**	To what extent is the following statement true? *…*<br>*E.g.* To what extent is the following statement true? Using glasses to see up close makes me feel older.	*a.* Not true at all.<br>*b.* Somewhat true.<br>*c.* Mostly true.<br>*d.* Completely true
**Convenience**	To what extent is the following statement true? *…*<br>*E.g.* To what extent is the following statement true? It takes me a while to put on and take off contact lenses.	*a.* Not true at all.<br>*b.* Somewhat true.<br>*c.* Mostly true.<br>*d.* Completely true
**Ocular surface symptoms**	*Have you…?*<br>*E.g. Have your eyes felt itchy?*	*a. Never*<br>*b. Rarely*<br>*c. Often*<br>*d. Always*
	Do you...?<br>*E.g. Do your eyes feel itchy?*	*a. No*<br>*b. Yes, a little*<br>*c. Yes, quite a bit*<br>*d. Yes, A lot*
	To what extent is the following statement true? *…*<br>*E.g. To what extent is the following statement true? I'm bothered by my eyes itching.*	*a.* Not true at all.<br>*b.* Somewhat true.<br>*c.* Mostly true.<br>*d.* Completely true

**General symptoms**	*Have you…?*<br>*E.g. Have you experiencing headaches?*	*a. Never*<br>*b. Rarely*<br>*c. Often*<br>*d. Always*
	Do you…?<br>*E.g. Does your head hurt?*	*a. No*<br>*b. Yes, a little*<br>*c. Yes, quite a bit*<br>*d.Yes, A lot*
	To what extent is the following statement true? …<br>*E.g.* To what extent is the following statement true? I'm bothered by having headaches	*a.* Not true at all.<br>*b.* Somewhat true.<br>*c.* Mostly true.<br>*d.* Completely true
**Visual Symptoms**	*Have you…?*<br>*E.g.* Have *you seen blurry?*	*a. Never*<br>*b. Rarely*<br>*c. Often*<br>*d. Always*
	Do you...?<br>*E.g. Do you see blurry?*	*a. No*<br>*b. Yes, a little*<br>*c. Yes, quite a bit*<br>*d. Yes, A lot*
	To what extent is the following statement true? …<br>*E.g.* To what extent is the following statement true? I'm bothered by seeing blurry.	*a.* Not true at all.<br>*b.* Somewhat true.<br>*c.* Mostly true.<br>*d.* Completely true
**Activity limitation**	To what extent is the following statement true? …<br>*E.g.* To what extent is the following statement true? I have difficulties when I work in a laptop.	*a.* Not true at all.<br>*b.* Somewhat true.<br>*c.* Mostly true.<br>*d.* Completely true<br>*e.* N/A

Table 2.

Item	Domain	BR	SM	PFG	OFG
How often have you found it difficult to keep appointments that are not part of your weekly routine?	Cognitive issues				did not come back for a check-up and came with the contact lenses from a year ago.
Are the products associated with the cleaning and care of your glasses or contact lenses an additional expense that you have been concerned about financially?	Economic Issues	How concerned are you about the cost of unscheduled maintenance of your contact lenses: breakage, loss, running out of supplies?		A contact lens is a daily expense	
Trying out new glasses or contact lenses excites me.	Emotional Well-Being			I never give up like that, at first time.	He wants them, and he wants them, he wants them, so in the end he adapts.
I have been conditioned to wear sunglasses.	Convenience	How concerned are you about eye protection from ultraviolet (UV) radiation?		The possibility of wearing any type of sunglasses	
How often have you experienced dry eyes?	Ocular symptoms	In the last week, how often did your eyes feel dry? (OCI)		In the morning I thought my eyes were drying up. They were like that, stuck together like chewing gum.	The main problem, I think, is the quality of the tear that we are encountering.
How problematic has it been for you to have a headache?	General symptoms		what a headache, it must be eyestrain	I wear them because otherwise I get a headache at the end of the day.	
How much have you felt your eyesight change during the day?	Visual Symptoms	how often have you experienced your vision changing/fluctuating throughout the day (CLIQ)		My eyesight also fluctuates, there are days when I see better than others and depending on the time of day.	
How much difficulty have you experienced in driving?	Activity limitation	How much difficulty do you have driving at night?		To get to the point that I can drive, without me running anyone over.	Having an active lifestyle, driving, computers, sport.
