# Supplementary material for: Content development for a new item-bank for measuring multifocal contact lens performance
Source: J Patient Rep Outcomes. 2024 Feb 8;8:16. doi: 10.1186/s41687-024-00689-w (PMC10853121; doi:10.1186/s41687-024-00689-w)
Supplement: Supplementary file 4 — Additional File 4: Item review guide. In this document you can read the guide followed for comprehensibility and comprehensiveness assessment. The content is in Spanish since the items are written in this language and it is the first language of the evaluators [file 41687_2024_689_MOESM4_ESM.pdf]

Guía de revisión de los ítems generados en la primera fase.

ESCUELA UNIVERSITARIA DE ÓPTICA (UNIVERSIDAD COMPLUTENSE)

Arcos de Jalón s/n 28037 Madrid
Tfno. 91 394 68 94 Fax 91 394 68 85

Estimados compañeros, como parte del desarrollo del cuestionario CVSS para la valoración de la sintomatología visual y ocular asociada al uso de videoterminales hemos solicitado vuestra colaboración para la primera evaluación del mismo, para lo cual necesitamos que evaluéis distintos aspectos formales del mismo. Os resumimos a continuación los criterios bajo los cuales debe realizarse la evaluación, estos criterios han sido extraídos del libro de Norman y Streiner[36] :

A.V.1. Redacción de los ítems

A.V.1.1. Claridad en la redacción:

La redacción de los ítems formulados en el cuestionario no debe ser difícil de comprender para el público general, aplicándose como regla práctica que el enunciado debe ser comprensible para un niño de 12 años.

Para la evaluación de este apartado hay que valorar cada ítem con una puntuación de 1 a 5, en donde 1 corresponde a un enunciado incomprensible, 3 a una redacción apropiada para un niño de 12 años y 5 a una redacción que podría comprender un niño muy pequeño.

1	3	5
Incomprensible	Nivel 12 años	Nivel 6 años

A.V.1.2. Longitud:

En general es preferible que los ítems sean lo más cortos posibles, aunque no tanto garantizando como para que pierdan comprensibilidad.

Para la evaluación de este apartado hay que valorar cada ítem con una puntuación de 1 a 5, en donde 1 corresponde a un enunciado demasiado extenso y 5 a un ítem de entre 10 y 20 caracteres.

1	3	5
Demasiado extenso	Extensión adecuada	10-20 caracteres

A.V.1.3. Ambigüedad:

Imaginemos un cuestionario diseñado para evaluar la atención hospitalaria en la que se formula un ítem compuesto de la raíz "Entendí la información que recibí por parte de:" tras la cual se detalla una lista de profesionales sanitarios y el entrevistado tiene que marcar "Sí" o "No". En este caso, una respuesta negativa en la opción "trabajador social" podría indicar que:

- El paciente no entendió lo que le dijo el trabajador social
- No le atendió ningún trabajador social
- No recuerda si le visitó o no el trabajador social

Aunque las dos últimas respuestas no entraban dentro de las opciones imaginadas al desarrollar el instrumento, la ambigüedad de la pregunto junto con el esquema de respuesta impuesto forzaron al sujeto a responder de forma ambigua.

También debe evitarse el uso de palabras como "a menudo", "últimamente" o "recientemente". Si se quiere preguntar acerca de un periodo de tiempo en concreto, se debe definir explícitamente.

Para la evaluación de este apartado hay que valorar cada ítem con una puntuación de 1 a 5, en donde 1 corresponde a un enunciado muy ambiguo y 5 a una redacción perfectamente clara.

1	3	5
Muy ambiguo	ni ambiguo ni claro	Perfectamente claro

A.V.1.4. Preguntas dobles:

Con pregunta doble nos referimos a aquellos ítems en que hacer dos preguntas a la vez, cada una de las cuales podría ser respondida de manera diferente. Este tipo de preguntas son muy frecuentes en los

cuestionarios sobre síntomas físicos y psicológicos, un ejemplo podría ser “Mis ojos se ponen rojos y llorosos”.

Algunas personas responden “sí” porque experimentan los dos síntomas, mientras que otros pueden responder afirmativamente aunque sólo experimenten uno de los dos con la consiguiente merma en la validez del ítem.

Un ejemplo más sutil de pregunta doble sería éste:

“No fumo por temor al cáncer de pulmón”

Verdadero □ Falso □

La gente que no fume por otros motivos se encontrará en una encrucijada a la hora de responder, ya que pueden no querer responder “Falso” porque esto implicaría que fuman y tampoco van a responder “Verdadero” porque esa no es la razón porque no fuman.

Para la evaluación de este apartado sólo hay que indicar si se trata de una pregunta doble (o múltiple) o no.

A.V.1.5. Jerga:

Los términos correspondientes a la jerga de nuestra profesión pueden colarse en un cuestionario de forma muy insidiosa. Dado que usamos de manera habitual un vocabulario fácilmente entendido por nuestros colegas, es fácil que lo utilicemos también con otros que no lo entiendan.

Algunas palabras pueden no ser entendidas como “diplopía”, otras pueden ser entendidas de forma errónea como “Tensión ocular”. Incluso palabras que los profesionales sanitarios creen que sus pacientes comprenden como “nutrición”, “digestión”, “por vía oral” o “tejido” se ha demostrado que en muchos casos no son entendidas correctamente.

Para la evaluación de este apartado sólo hay que indicar si el ítem evaluado contiene vocabulario propio de la jerga de los profesionales sanitarios o no.

A.V.1.6. Preguntas que predisponen a la respuesta:

La inclusión de determinadas palabras en la pregunta puede predisponer a los entrevistados a responder en un determinado sentido, podemos citar como ejemplo los siguientes ítems:

"¿Va a menudo al médico por problemas triviales?"

"¿Piensa que los dentistas ganan mucho dinero?"

ó

"Dado que [cierto producto] ahorraría mucho dinero a su hospital, ¿estaría dispuesto a utilizar [el mencionado producto] en la cirugía de cataratas?"

Para la evaluación de este apartado sólo hay que indicar si la formulación del ítem predispone a responder en un cierto sentido o no.

A.V.1.7. Impresión personal:

En esta parte hay que valorar de 1 (muy mala impresión) a 5 (muy buena impresión) la impresión que el evaluador tiene de este ítem y de la conveniencia de incluirlo en el cuestionario

A.V.2. Evaluación:

Junto con la guía de revisión hemos enviado un archivo Excel en el que se ha escrito cada ítem de los generados en la primera fase del proyecto. Como se puede apreciar en la siguiente imagen, el evaluador debe leer cada ítem y evaluar en las casillas situadas a la derecha cada uno de los aspectos a valorar de los ítems.

	Claridad	Longitud	Ambigüedad	Pregunta Doble	Jerga	Predispone
¿Cuánto tiempo puedes realizar trabajo con el ordenador (leer, escribir,navegar...) sin notar ninguna molestia en los ojos o cerca de ellos (dolor de cabeza, de ojos, ardor, visión doble o borrosa, lagrimeo, escozor...?	3	2	4	si	no	no
¿Con que frecuencia tiene dolores de cabeza mientras trabaja con su ordenador?	5	4	5	no	no	no
Si tiene dolores de cabeza durante el trabajo con su ordenador ¿Cuánto de fastidiosos diría que son?	3	4	4	si	no	no
¿Con que frecuencia nota en sus ojos sensación de tirantez, lagrimeo o dolor mientras que trabaja con su ordenador?	4	3	5	si	no	no
¿Ha notado que a veces se le emborronan las letras del ordenador mientras trabaja con él?	3	4	4	no	no	no
¿Ha notado que a veces se juntan, se amontonan o se mueven las letras del ordenador mientras trabaja con él?	5	4	5	si	no	no
